# Microvascular endothelial metabolic dysfunction drives cerebral edema through bioenergetic failure after ischemia-reperfusion

**DOI:** 10.7150/thno.127083

**Published:** 2026-01-01

**Authors:** Yi-Fan Zhou, Si-Bo Yang, Feng Zhang, Xiao-Di Sun, Bo-Hao Chang, Ya-Nan Li, Jie-Hong Wu, Hui-Juan Jin, Ming Huang, Sheng-Cai Chen, Hang Yang, Dong-Ya Zhu, Bijoy K Menon, Bo Hu

**Affiliations:** 1 Department of Neurology, Union Hospital, Tongji Medical College, Huazhong University of Science and Technology, Wuhan 430022, China.; 2 Department of Neurology, Hubei Provincial Hospital of Integrated Chinese and Western Medicine, Hubei University of Chinese Medicine, Wuhan, China.; 3 Department of Clinic Pharmacology, School of Pharmacy, Nanjing Medical University, Nanjing 211166, China.; 4 Department of Community Health Sciences, Department of Clinical Neurosciences, Department of Radiology, and Hotchkiss Brain Institute, University of Calgary, AB, Canada.

**Keywords:** lactate metabolism, histone lactylation, endothelial bioenergetic failure, necroptosis, cerebral edema after endovascular recanalization therapy

## Abstract

**Aim:** Despite major advances in recanalization therapy, poor functional outcomes after ischemic stroke remain common. The central challenge is not only restoring blood flow but also overcoming the bioenergetic failure that can persist during reperfusion. This study aims to identify the missing link by defining how a metabolic-epigenetic cascade drives microvascular energetic collapse, thereby elucidating mechanisms underlying sustained cerebral edema and the no-reflow phenomenon following ischemia-reperfusion.

**Methods:** We analyzed serum samples from patients with acute ischemic stroke to evaluate associations among lactate/pyruvate (L/P) ratios, functional outcomes, and cerebral edema. High-resolution magnetic resonance imaging (MRI) and FITC-dextran extravasation test in transient middle cerebral artery occlusion (tMCAO) model were used to determine whether glycolytic inhibition reduced edema. Single-cell RNA sequencing characterized endothelial cell subpopulations after stroke, and molecular experiments examined the effects of lactate accumulation on histone H3K18 lactylation (H3K18la) and downstream ATF4-DDIT4 signaling. Mitochondrial function, electron transport chain (ETC) activity, and necroptosis-related pathways were assessed in endothelial cells.

**Results:** An elevated L/P ratio was strongly correlated with poor neurological outcomes and was closely linked to ischemia-reperfusion-induced cerebral edema. Reducing glycolytic flux and lactate production attenuated edema formation. Single-cell sequencing identified a post-stroke hyper-glycolytic endothelial subset characterized by mitochondrial dysfunction and necroptosis activation, with greater expansion in aged tMCAO mice. Lactate accumulation increased H3K18la in endothelial cells and activated the ATF4-DDIT4 pathway, which further impaired mitochondrial and ETC function. These changes established a self-amplifying pathological loop —glycolysis/H3K18la/ATF4-DDIT4 — that intensified bioenergetic failure while promoting RIPK3-dependent necroptosis and inflammation.

**Conclusions:** Aberrant lactate metabolism not only serves as a prognostic biomarker but also provides a mechanistic link between metabolic insufficiency and epigenetic dysregulation through histone lactylation. Targeting the feedback loop involving H3K18la and ATF4-DDIT4 offers a promising therapeutic approach to limit cerebral edema and improve functional outcomes after ischemic stroke.

## Introduction

Ischemic stroke is a major public health concern because of its high incidence, mortality, and recurrence. Timely vascular recanalization via intravenous thrombolysis or endovascular thrombectomy is the standard of care. However, clinical outcomes often reveal a therapeutic paradox. Despite recanalization success rates of 70-90%, more than 50% of patients with restored blood flow still experience poor functional outcomes^1^.

This disconnect between macrovascular success and microvascular failure highlights a central limitation of current stroke therapy. Clinically ineffective reperfusion, characterized by persistent cerebral edema, no-reflow, and reperfusion injury^2^, underscores that restoring blood flow alone often fails to resolve the underlying metabolic crisis^3^. Reperfusion may further exacerbate metabolic dysfunction, manifesting as systemic hyperglycemia and impaired cerebral glucose utilization^4^, ^5^. Restoration of metabolic homeostasis after reperfusion is a complex and dynamic process rather than a binary switch. Notably, significant glycemic variability after thrombectomy serves as an indicator of reperfusion injury and is associated with poor prognosis^6^. Thus, the degree of metabolic restoration is a key determinant of both cellular survival and clinical outcomes. Understanding how bioenergetic failure governs cell fate under ischemia-reperfusion stress is crucial for developing precise early-warning and risk-prediction strategies.

Advanced age further amplifies these metabolic disturbances^7^. Aging accelerates early edema progression and worsens blood-brain barrier (BBB) disruption after ischemic injury^8^, in part due to altered metabolic reprogramming that compromises endothelial function^9^, ^10^. Although glycolysis inhibition has shown therapeutic potential in other neurological disorders^11^, ^12^, its role in stroke remains underexplored.

Endothelial cells (ECs), which are critical regulators of BBB integrity, depend heavily on glycolysis to meet energy demands^13^. Although acute glycolytic activation may support adaptive responses, sustained hyper-glycolysis triggers mitochondrial dysfunction, excessive reactive oxygen species (ROS) production, and accelerated cellular senescence and death^14^. Ischemic stress elevates lactate production, creating a microenvironment that promotes lactate accumulation within ECs. Beyond its metabolic role, lactate functions as a signaling molecule that modulates EC metabolism and barrier function^15^. Notably, lactate-induced histone lactylation can directly regulate gene transcription^16^, and has been implicated in pathological endothelial reprogramming, including promotion of atherosclerosis via endothelial-to-mesenchymal transition (EndMT)^17^.

Here, we found that elevated serum lactate and increased glycolytic activity in stroke patients were associated with poor prognosis and aggravated cerebral edema after recanalization. We observed an expansion of a hyper-glycolytic endothelial subpopulation that was particularly pronounced in aged mice and was characterized by mitochondrial dysfunction and necroptosis activation. Mechanistically, lactate-driven H3K18la epigenetically disrupts mitochondrial and ETC function through the ATF4-DDIT4 pathway, leading to bioenergetic failure and RIPK3-dependent necroptosis. These findings establish a pathogenic glycolysis/H3K18la/ATF4-DDIT4 feedback loop that sustains BBB injury post-reperfusion. Early detection of increased lactate and glycolytic activity may enable personalized interventions targeting this metabolic-epigenetic axis, thereby mitigating BBB damage and improving neurological outcome.

## Results

### Elevated serum lactate levels are associated with poor prognoses

**Figure 1:**
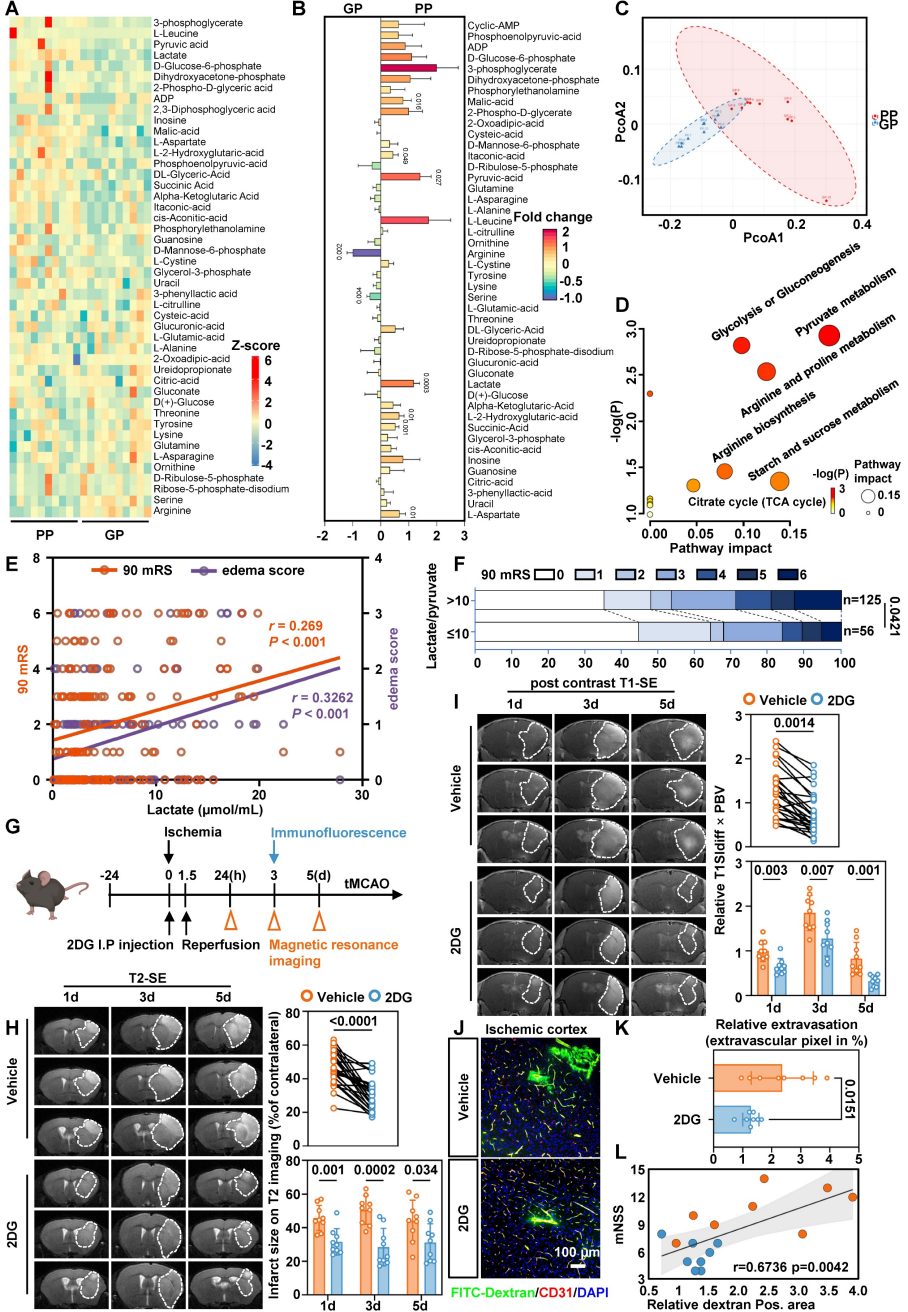
** Serum lactate is elevated in patients with a poor prognosis and inhibition of glycolytic pathway alleviates edema and BBB injury. A,** Heatmap of altered metabolites from targeted energy metabolite profiling between the PP (left) and GP (right) group. Color scale indicates Z-score: Z-score > 0 are upregulated (red); Z-score < 0 are downregulated (blue). **B,** Fold change plot of detected metabolites from targeted metabolite profiling of GP and PP serum. Color scale indicates the degree of log2 fold change (red, upregulated > 2; violet, downregulated < -1). **C,** PCA plot of the PC 1 and 2 of significantly altered metabolites from ten GP and ten PP serum samples. **D,** Pathway matches of significantly changed metabolites in PP serum compared to GP serum based on targeted metabolite profiling. Circle size reflects the pathway impact and color code visualizes -log(p) value (white = 0, yellow = 1, orange = 2, red > 3). **E,** Three-factor correlation analysis involving the patient's 90-day mRS, serum lactate levels, and edema score. **F,** The modified Rankin Scale was employed to assess the clinical progression of patients at 90 days, with scores ranging from 0 to 6. Specifically, a score of 0 indicates the absence of symptoms, 1 signifies no clinically significant disability, 2 denotes mild disability, 3 reflects moderate disability, 4 represents moderately severe disability, 5 indicates severe disability, and finally, a score of 6 corresponds to death. These figures illustrate the proportion of patients within each category (%). **G,** Experimental design and timeline for 2DG treatment, and analysis in tMCAO mice. tMCAO refers to transient middle cerebral artery occlusion. **H,** Representative MRI T2 images. Statistical brain infarct volume which was detected using T2-weighted MRI at 1,3,5 days after tMCAO (the upper n = 27 per group and the lower n = 9 per group). **I,** Representative MRI post-contrast T1 images. White dashed circles represent the leakage area. Quantification of BBB permeability by T1SIdiff ✕ PBV (the upper n = 27 per group and the lower n = 9 per group). **J.** Representative images of immunofluorescence staining of FITC-Dextran and endothelial marker CD31 in ischemia-reperfusion cortex. **K.** Quantification of relative extravasation of FITC-dextran in tMCAO mice (n = 8 per group). **L.** Analysis correlation between Dextran area and mNSS score. Data are mean ± SEM.

To characterize metabolic alterations, we analyzed 20 patients older than 65 years from the TRAIS cohort and stratified them into poor prognosis (PP) and good prognosis (GP) groups based on 90-day mRS and edema scores. Targeted serum metabolomics detected 46 metabolites, which were visualized as a heatmap (Figure 1A). Nine metabolites differed significantly between groups (Figure 1B). Principal component analysis (PCA) clearly distinguished PP from GP patients based on their metabolic profiles (Figure 1C). Notably, lactate levels were higher in the PP group, consistent with increased glycolytic activity (Figure 1D).

In a larger cohort of 181 patients, colorimetric analysis showed that serum lactate levels above 2.2 µmol/mL were associated with a 2.247-fold increased risk of residual neurological disability at 90 days [95% CI, 1.166-4.33] and a 2.659-fold higher risk of cerebral edema [95% CI, 1.38-5.123] (Table 1 and Table S1). Three-factor correlation analysis further supported the association among serum lactate, cerebral edema, and poor outcomes (Figure 1E).

To better capture the bioenergetic status of human, we introduced the L/P ratio. The L/P ratio reflects the intracellular redox state, and an elevated ratio indicates a bioenergetic shift from oxidative phosphorylation to glycolysis. The L/P ratio also showed similar trends (Table 3), with the most pronounced alterations observed in elderly patients (Table 2 and Table 4). Follow-up after recanalization demonstrated a positive correlation between the L/P ratio and 90-day mRS scores (Figure 1F). Together, these findings suggest that a bioenergetic shift toward glycolysis is closely associated with adverse prognosis and cerebral edema, and that serum lactate and L/P ratio may serve as potential biomarkers.

### Inhibiting glycolysis reduces lactate production alleviates edema and BBB injury in tMCAO mice

Based on these findings, we investigated whether reducing lactate levels mitigates cerebral edema post-ischemia-reperfusion. We administered 2-deoxy-D-glucose (2-DG), a glycolytic inhibitor, via intraperitoneal injection in a tMCAO model and focused on endothelial injury during reperfusion (Figure 1G). No toxicity or mortality was observed in aged mice prior to MCAO. In tMCAO mice, T2-weighted MRI showed that 2-DG treatment significantly reduced infarct volume (Figure 1H and Supplementary Figure S1A), while quantification of T1-weighted MRI following contrast agent administration showed that 2-DG treatment alleviated cerebral edema (Figure 1I and Supplementary Figure S1B). Consistently, immunofluorescence staining with FITC-dextran showed a significant reduction in the leakage area in the ischemia-reperfusion cortex (Figure 1J-L).

### BMEC glycolysis is enhanced after stroke in tMCAO mice

**Figure 2:**
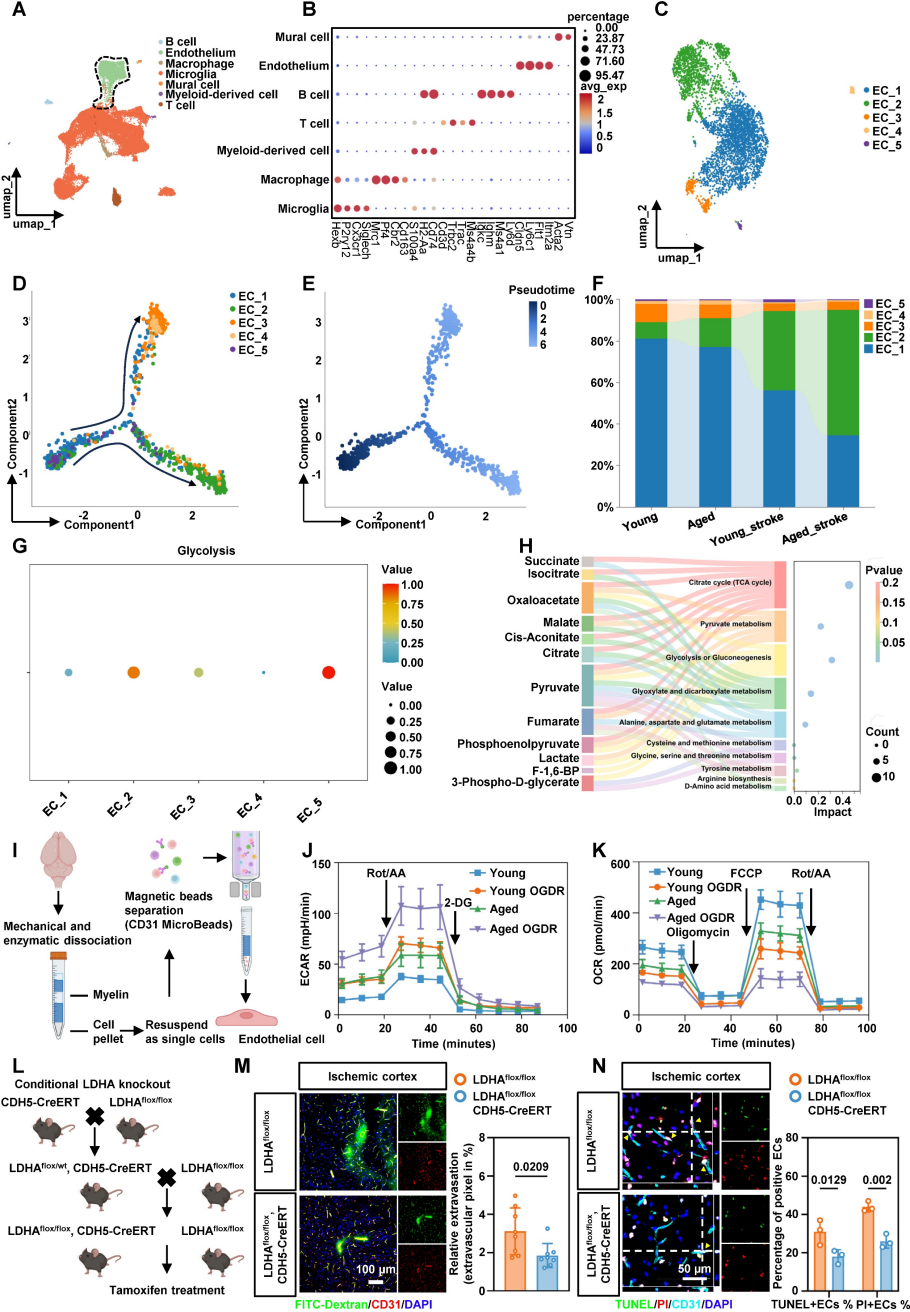
** Endothelial cell glycolysis is upregulated after stroke in tMCAO mice. A,** Clustering of scRNA-seq data was performed. Cell types were annotated based on the expression levels of known marker genes. **B,** The expression profiles of marker genes for each cell type were analyzed. **C,** UMAP plot showing populations of re-clustered ECs. **D-E,** Pseudo-time analysis illustrating the developmental trajectory of spots derived from ECs, distinguished by clusters, states, and pseudo-time. **F,** The percentage accumulation bar chart illustrates the relative distribution of each subcluster within disease models involving aged and young mice. **G,** Dot plot showing glycolytic metabolic pathways in each cluster of ECs. **H,** Sankey bubble chart of energy metabolomics analysis of microvascular tissues in tMCAO mice. **I,** Schematic of endothelial cell isolated from adult brains of 8-week-old mice and 20-month-old mice. **J,** The ECs isolated from the brains of aged and young mice were treated with OGDR, after which the ECAR was measured to quantify the glycolysis rate (n = 5 mice per group). **K,** The ECs isolated from the brains of aged and young mice were treated with OGDR. Subsequently, the OCR was measured to quantify basal respiration, ATP-linked respiration, and maximal respiration (n = 5 mice per group). **L.** The schematic diagram showing the breeding strategy for generating mice which were specifically lacking LDHA expression in endothelial cell (LDHA^flox/flox^; CDH5-CreERT). **M.** Representative images of immunofluorescence staining of FITC-Dextran and endothelial marker CD31 in ischemia-reperfusion cortex and quantification of relative extravasation of FITC-dextran in the cortex of LDHA^flox/flox^ or LDHA^flox/flox^; CDH5-CreERT mice following ischemia-reperfusion (n = 8 per group). **N.** Representative images and statistical analysis results of immunofluorescence staining for TUNEL (green, indicating apoptosis) and PI (red, indicating necrosis) in ECs within the cortical area of LDHA^flox/flox^ or LDHA^flox/flox^; CDH5-CreERT mice (n = 3 per group). Data were used the two-factor ANOVA of statistical methods (**j, k**). Data are mean ± SEM.

Given the trend of worsened cerebral edema in patients and the possibility that aging exacerbates this phenotype, we focused on brain microvascular endothelial cells (BMECs) and reanalyzed publicly available single-cell RNA sequencing (scRNA-seq) data from young and aged mice^18^. We annotated all cell populations based on canonical marker gene expression (Figure 2A-B). Secondary clustering of ECs identified five phenotypically distinct subtypes (Figure 2C). Pseudotime trajectory analysis revealed dynamic shifts in EC phenotypes over time, which were visualized by cluster identity, cell state, and pseudotime progression (Figure 2D-E). After ischemia-reperfusion, the proportion of ECs in cluster 2 increased significantly (Figure 2F). We next used the R package scMetabolism to infer metabolic activity in individual ECs. Single-cell analysis showed a pronounced upregulation of glycolysis in cluster 2 ECs following ischemic stroke (Figure 2G). Gene Ontology (GO) enrichment of differentially expressed genes (DEGs) in this cluster highlighted pathways related to canonical glycolysis, mitophagy, and mitochondrial apoptosis (Figure S2A). KEGG pathway analysis further identified HIF-1, PI3K-Akt, and necroptosis signaling (Figure S2B). Collectively, cluster 2 ECs represent a hyper-glycolytic population characterized by mitochondrial dysfunction and increased susceptibility to necroptotic cell death.

Energy metabolomics of microvascular tissue from tMCAO mice confirmed significant lactate accumulation after ischemia-reperfusion (Figure 2H). Colorimetric assays in both aged and young mice corroborated these findings (Figure S2C). To examine endothelial energy metabolism in greater detail, BMECs were isolated from 8-week-old and 20-month-old mice using magnetic separation (Figure 2I). Extracellular acidification rate (ECAR) measurements showed that aged ECs exhibited higher baseline glycolytic capacity than young ECs, indicating an age-dependent enhancement of glycolytic metabolism under ischemic stress. After ischemic treatment with oxygen-glucose deprivation and reperfusion (OGDR), glycolytic capacity increased significantly in ECs from both young and aged mice (Figure 2J, Figure S2D-E). In contrast, oxygen consumption rate (OCR) measurements indicated that ECs from aged mice had markedly impaired mitochondrial function compared to young mice. Aged ECs also exhibited greater vulnerability to ischemia-reperfusion injury (Figure 2K, Figure S2F-H). Together, these results indicate that ECs undergo a substantial bioenergetic shift from oxidative phosphorylation to glycolysis following ischemia-reperfusion, and this shift is more pronounced in aged mice.

### Conditional knockout of LDHA in BMECs alleviates BBB injury and EC death

To suppress the abnormally active glycolysis in ECs, we generated endothelia-specific LDHA-deficient mice by crossing LDHA^flox/flox^ mice with CDH5-CreERT mice (Figure 2L). LDHA expression was effectively reduced in LDHA^flox/flox^; CDH5-CreERT mice (Figure S3A, B). LDHA^flox/flox^; CDH5-CreERT mice and their LDHA^flox/flox^ littermates were subjected to tMCAO. Immunofluorescence staining with FITC-dextran in the ischemia-reperfusion cortex demonstrated an improved BBB permeability in LDHA^flox/flox^; CDH5-CreERT mice (Figure 2M). In addition, dual staining with TUNEL and propidium iodide (PI) showed reduced apoptosis and necrosis in BMECs from LDHA^flox/flox^; CDH5-CreERT mice (Figure 2N). Transmission electron microscopy (TEM) further validated that LDHA knockout restored mitochondrial morphology and structure in BMECs from ischemia-reperfusion region (Figure S3C). Collectively, these results suggest that inhibiting abnormally active glycolysis after ischemia-reperfusion reduces BMEC death and alleviate BBB injury in tMCAO mice.

### Ischemia-reperfusion alters the histone lactylation profile of BMECs in tMCAO mice

**Figure 3:**
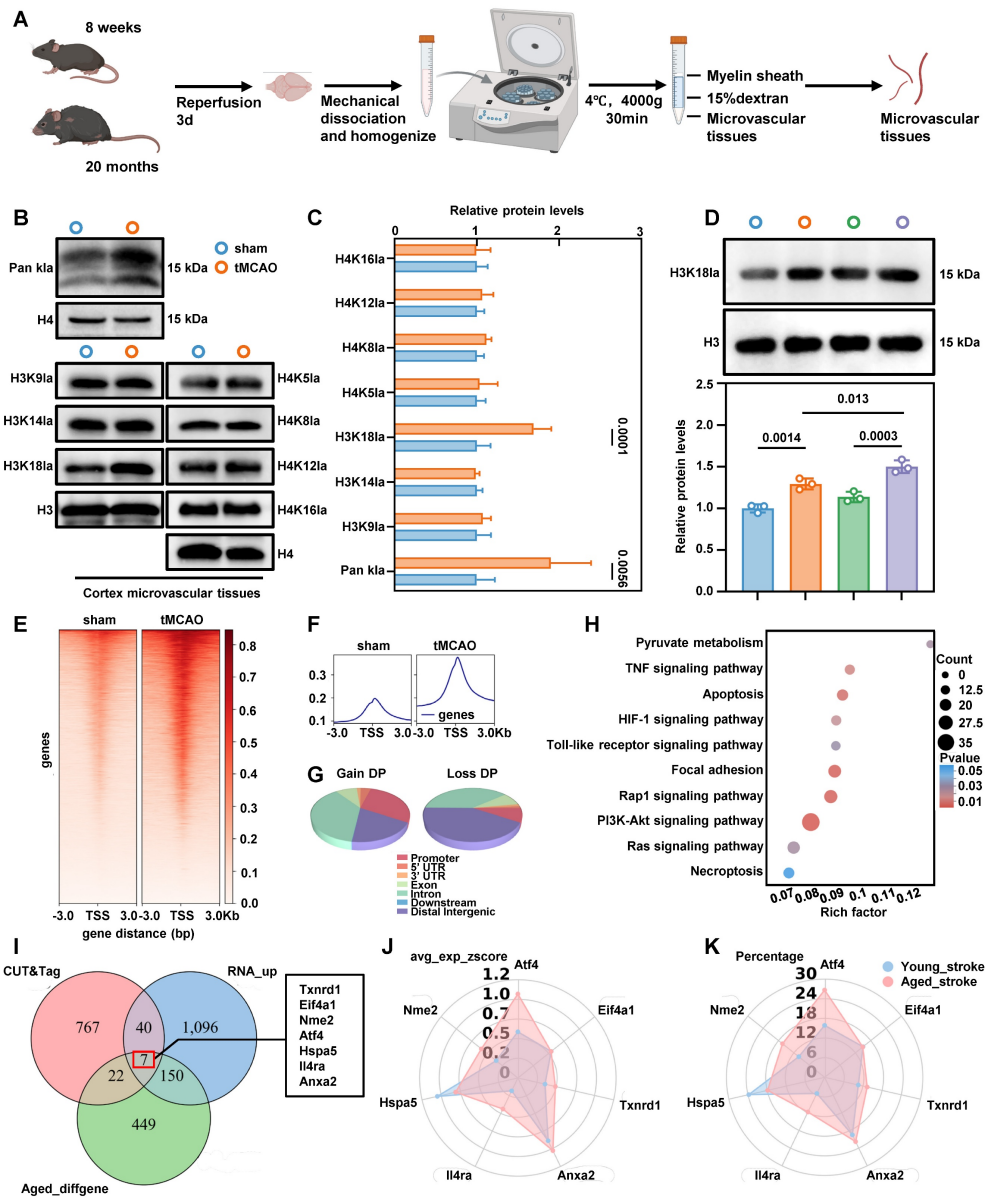
** Genome-wide analysis of the transcriptional consequences of H3K18la in endothelial cell. A,** Schematic representations of the separation of brain microvascular segments from aged and young mice. **B-C,** Western blotting analysis of Pan- and site-specific histone lactylation in the microvascular segments of sham operation group and ischemia-reperfusion mice, with quantification of protein levels (n = 5-6 per group). **D,** Western blot analysis of H3K18la in microvascular tissues before and after ischemia-reperfusion in 8-week-old mice and 20-month-old mice, with quantification of protein levels (bottom panel, n = 3 per group). **E-F,** The distribution of CUT&Tag signals for H3K18la in proximity to the transcription start site. **G,** Genome-wide distribution of differentially H3K18la-binding peaks in microvascular tissues from tMCAO mic. **H,** KEGG analysis of the elevated H3K18la binding peaks at candidate target genes. **I,** Overlapping analysis to identify potential targets of H3K18la within the transcriptomic landscape during ischemia-reperfusion in aged mice. RNA-UP refers to the gene whose expression significantly increases following ischemia-reperfusion in aged mice, as identified through RNA sequencing (RNA-seq). CUT&Tag highlights genes that exhibit significant differences in H3K18la binding. Old_diffgene denotes the DEGs observed before and after ischemia-reperfusion in elderly mice, based on single-cell sequencing results. **J-K,** The radar plot provides a comprehensive analysis of the expression changes of the aforementioned genes before and after ischemia-reperfusion in both old and young mice, encompassing percentage values and average expression z-scores. Data are mean ± SEM.

Given that lactate acts as a substrate for histone modification^16^, and our data show that lowering lactate levels reduces BBB injury, we next examined changes in histone lactylation in BMECs under ischemia-reperfusion conditions. We isolated brain microvascular tissues using density gradient centrifugation (Figure 3A), and microvessel purity was verified by immunofluorescence staining (Figure S3D). Western blot analysis of acid-extracted histones from these tissues showed a significant increase in pan-lysine lactylation and H3K18la in BMECs from tMCAO mice (Figure 3B-C). Compared with young mice, aged mice exhibited a more pronounced elevation in H3K18la levels in microvascular tissues following ischemia-reperfusion (Figure 3D). Notably, H3K18la and lactate levels in microvascular tissues were positively correlated with serum lactate concentrations (Figure S4A).

To investigate the potential functional role of H3K18la in BMECs, we performed genome-wide profiling of this mark in aged stroke model mice. We collected microvascular tissues from 20-month-old mice subjected to either sham surgery or tMCAO. CUT&Tag using H3K18la-specific antibodies, followed by deepTools analysis, revealed a marked enrichment of H3K18la peaks in the microvascular tissues of ischemia-reperfusion brains compared to sham controls (Figure 3E-F). Differential signal analysis in open chromatin regions identified 2183 distinct binding peaks in the ischemia-reperfusion group compared to the sham group. Among these, 1610 peaks showed significant upregulation, and 28.84% were located in promoter regions (≤ 3kb from the transcription start site) (Figure 3G). We annotated genes associated with these promoter-bound H3K18la peaks in microvascular tissues from tMCAO-aged mice and classified them using GO analysis (Figure 3H). KEGG pathway enrichment highlighted HIF-1, PI3K-Akt, TNF-signaling, and necroptosis pathways, which are closely-related to glycolysis and cell death. These findings demonstrate that both lactate levels and histone lactylation increase following ischemia-reperfusion, with H3K18la emerging as the most significantly altered histone lactylation mark in BMECs from aged stroke model mice.

### Enhanced H3K18la induces ATF4 expression in BMECs

To investigate H3K18la-mediated epigenetic regulation following ischemia-reperfusion in aged mice, we performed an integrated analysis of RNA sequencing, H3K18la CUT&Tag, and scRNA-seq data. This analysis identified seven genes that were significantly upregulated by H3K18la and showed notable expression differences before and after ischemia-reperfusion in aged mice (Figure 3I). Further analysis of single-cell datasets from both aged and young mice revealed that, among these candidates, the transcription factor ATF4 exhibited the most pronounced expression changes in aged mice following ischemia-reperfusion (Figure 3J-K). Consistently, H3K18la enrichment at the ATF4 promoter increased after ischemia-reperfusion (Figure S4B). Quantitative chromatin immunoprecipitation (qChIP) analysis confirmed a significant elevation of H3K18la occupancy at the ATF4 promoter in microvascular tissues from aged mice subjected to ischemia-reperfusion, compared to sham controls (Figure S4C). Furthermore, primary microvascular ECs overexpressing LDHA, which boosts endogenous lactate production, exhibited increased ATF4 expression (Figure S4D-F). Together, these findings demonstrate that the ATF4 is transcriptionally upregulated in BMECs after ischemia-reperfusion and that this induction is associated with H3K18la modification.

### ATF4 promotes DDIT4 transcription and EC necroptosis

**Figure 4:**
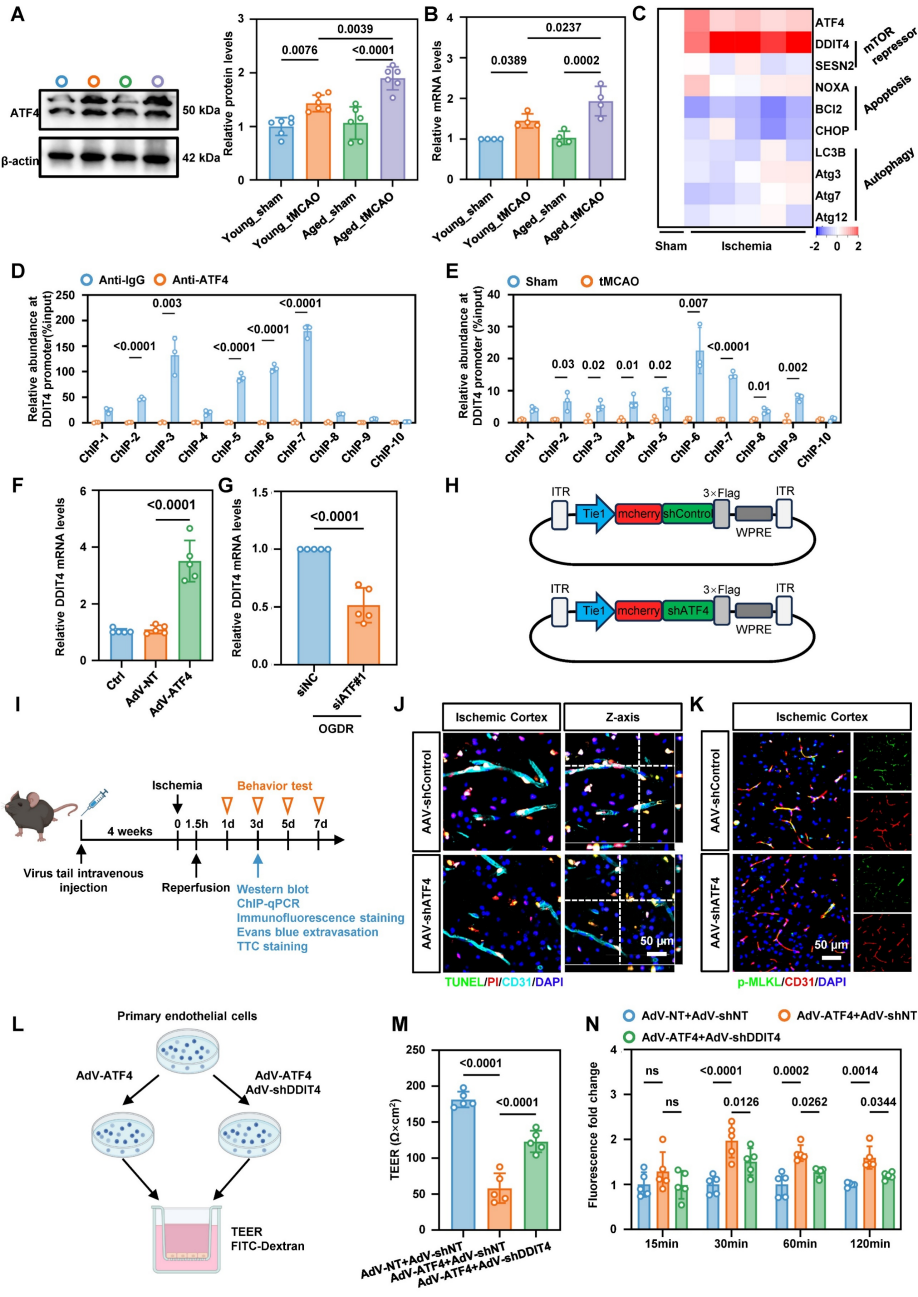
** ATF4 promotes DDIT4 transcription leads to EC necroptosis and BBB injury in tMCAO mice. A,** Western blot analysis of ATF4 protein levels in brain microvascular tissues isolated from 8-week-old and 20-month-old tMCAO mice, with quantification of protein levels (right panel, n = 6 per group). **B,** Real-time quantitative PCR analysis of ATF4 mRNA levels in brain microvascular tissues isolated from 8-week-old and 20-month-old tMCAO mice (n = 4 per group). **C,** Heat map illustrates the mRNA expression levels of key target genes associated with ATF4 transcriptional activation in the microvascular tissues of aged mice. Values were determined by qRT-PCR and plotted as fold change relative to control.** D-E,** qChIP analysis of the DDIT4 promoters was performed using antibodies against ATF4 in microvascular tissues from aged mice after ischemic-reperfusion (n = 3 per group). **F,** Changes in the mRNA levels of the downstream target gene DDIT4 were observed following ATF4 overexpression (n = 5 per group). **G,** Changes in the mRNA levels of the downstream target gene DDIT4 were observed following simultaneous ATF4 knockdown during *in vitro* oxygen-glucose deprivation and reperfusion (n = 5 per group). **H.** Schematic diagram of the AAV used for ATF4 knockdown *in vivo*. **I,** Experimental design and timeline of ischemic stroke, ATF4 knockdown, and analysis in aged mice. **J,** Representative images results of immunofluorescence staining for TUNEL (green, indicating apoptosis) and PI (red, indicating necrosis) in ECs within the ischemia-reperfusion cortical region following ATF4 knockdown. **K,** Representative images illustrating the co-staining of p-MLKL with BMECs (CD31) of the ischemia-reperfusion in cortical region following ATF4 knockdown. **L,** Illustration of TEER permeability assay and FITC-dextran permeability assay. **M-N,** TEER and FITC permeability of vehicle-treated (AdV-shNT) versus AdV-shDDIT4 treated ECs under ATF4 overexpression. (n = 5 independent experiments). Data are mean ± SEM.

To further examine the effect of aberrant ATF4 expression on BMECs in tMCAO mice, we measured ATF4 levels in isolated microvascular tissues by western blotting and qPCR. Both assays revealed a significant increase in endothelial ATF4 accumulation in aged tMCAO mice compared to their younger counterparts (Figure 4A-B). ATF4 is a transcription factor, that regulates apoptosis, cell cycle arrest, and cellular senescence, and it also contributes to the balance between autophagy and protein synthesis^19^. To further investigate the downstream targets of ATF4, we measured the expression of representative ATF4 responsive genes in cerebral microvascular tissues of aged mice by qPCR. Our data showed that increased ATF4 expression in aged ECs following ischemia-reperfusion was accompanied by higher expression of the mTOR repressor DDIT4 (Figure 4C). As a stress-responsive protein, DDIT4 is involved in modulating energy balance and metabolism, and has been proposed as a potential therapeutic target for treatment of metabolic disorders^20^. qChIP results confirmed a physical interaction between the promoters of ATF4 and DDIT4 (Figure 4D), and ischemia-reperfusion in cerebral microvascular tissues from tMCAO mice further increased ATF4 binding at the DDIT4 promoter region (Figure 4E). Primary BMECs were transfected *in vitro* with siRNA or adenoviruses to achieve ATF4 knockdown or overexpression (Figure S5A-B). Consistent with the *in vivo* findings, both ATF4 knockdown and overexpression modulated DDIT4 transcriptional levels (Figure 4F-G).

To assess the functional role of ATF4* in vivo*, we employed an adeno-associated virus (AAV)-mediated knockdown strategy. CNS microvasculature EC-targeted AAV-BR1 was used to deliver shRNA against ATF4 transcripts and achieve gene silencing in the brain vasculature (Figure 4H). 20-month-old mice received AAV-shControl or AAV-shATF4 by tail vein injection. Four weeks later, ischemia was induced by middle cerebral artery occlusion followed by reperfusion, as shown in the experimental workflow (Figure 4I). Knockdown efficiency was confirmed by western blotting and confocal microscopy (Figure S6A-B). Immunofluorescence staining of PI and TUNEL in anti-CD31 positive ECs revealed that ATF4 knockdown reduced ischemia-reperfusion-induced BMEC death (Figure 4J, Figure S6C). ATF4 knockdown also reduced p-MLKL expression in BMECs based on immunofluorescence staining and quantitative analysis (Figure 4K, Figure S6D). To evaluate outcomes after ischemia-reperfusion outcomes, we performed Evans blue staining, 2,3,5-triphenyltetrazolium chloride (TTC) staining, and neurological severity scoring. Quantitative analyses showed that ATF4 downregulation in BMECs significantly alleviated BBB injury, reduced infarct volume, and improved neurological performance (Figure S6E-G).

To clarify the role of the ATF4-DDIT4 axis in BBB injury, we performed a trans-endothelial electrical resistance (TEER) assay and measured paracellular flux of dextran particles (Figure 4L). Primary BMECs transfected with adenoviruses to achieve DDIT4 knockdown or overexpression (Figure S7A-B). These experiments demonstrated that ATF4-DDIT4 signaling impaired barrier function and that DDIT4 knockdown attenuated this effect (Figure 4M-N).

### Conditional knockdown of DDIT4 in BMECs reduces BBB injury and EC necroptosis post-ischemia-reperfusion

**Figure 5:**
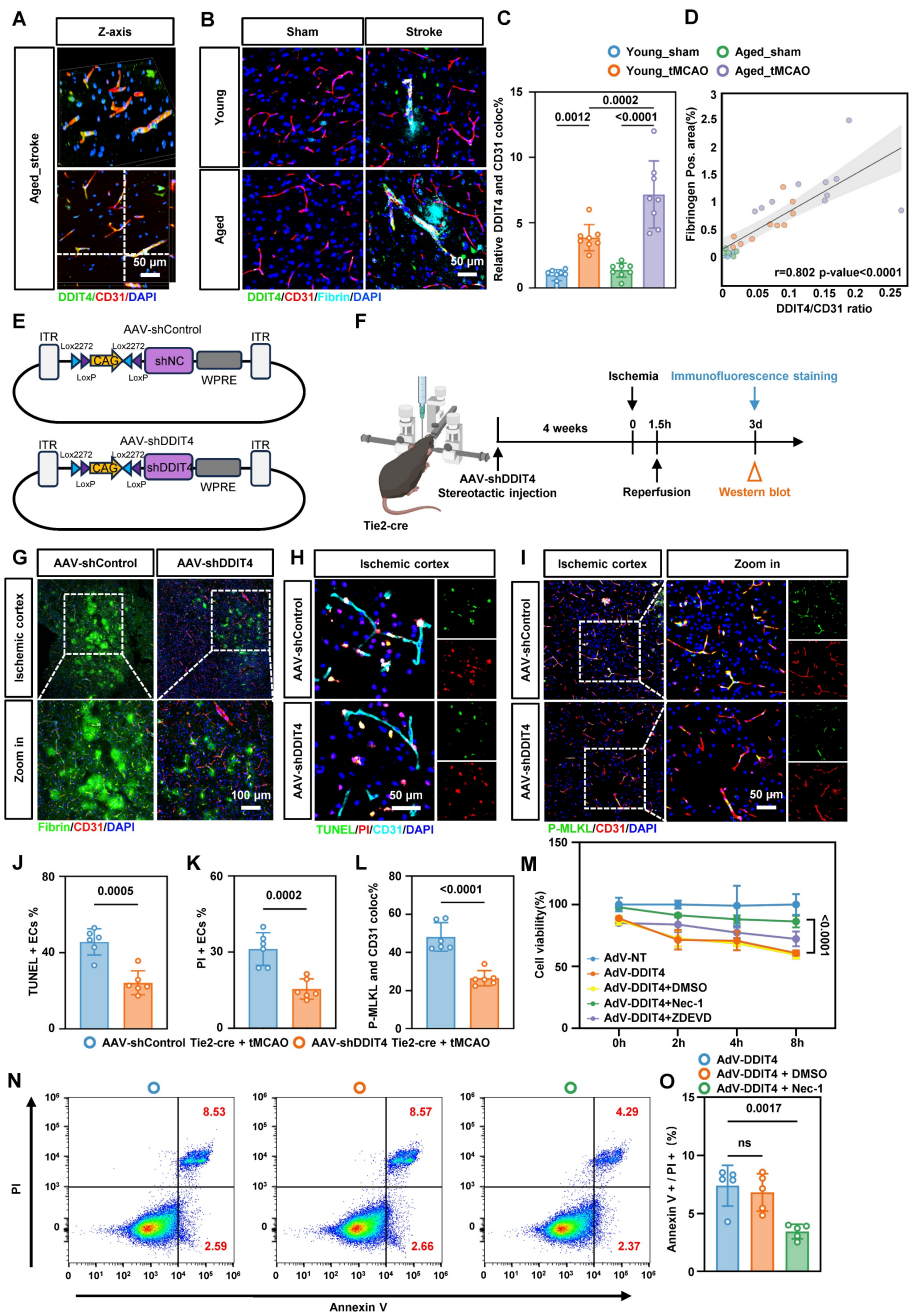
** Genetic knockdown of DDIT4 ameliorates EC necroptosis and BBB injury in tMCAO mice. A,** Representative images of reconstructed Z-stack showing colocalization of DDIT4 and endothelial cell marker CD31 in the cortex of tMCAO mice. **B,** Representative images co-stained with fibrin, DDIT4 and cortical microvascular ECs (CD31) in the ischemia-reperfusion area. **C,** Quantitative analysis of the co-localization of DDIT4 and ECs (n = 8 per group). **D,** Analysis of correlation between co-localization ratio of DDIT4 and CD31 (represented by DDIT4/CD31) and Fibrinogen area. **E,** Schematic diagram of the AAV used for DDIT4 knockdown *in vivo*. **F,** Experimental design and timeline of ischemic stroke, DDIT4 knockdown, and analysis in aged mice. **G,** Representative images co-stained with fibrin and cortical microvascular ECs (CD31) in the ischemia-reperfusion area from DDIT4 conditional knockdown mice. **H,** Representative images results of immunofluorescence staining for TUNEL (green, indicating apoptosis) and PI (red, indicating necrosis) in ECs within the ischemia-reperfusion cortical region following DDIT4 knockdown. **I,** Representative images illustrating the co-staining of p-MLKL with BMECs (CD31) of the ischemia-reperfusion in cortical region following DDIT4 knockdown. **J-K,** Statistical analysis results of immunofluorescence staining for TUNEL (green, indicating apoptosis) and PI (red, indicating necrosis) in ECs within the ischemia-reperfusion cortical region following DDIT4 knockdown (n = 6 per group). **L,** Quantitative analysis of the co-localization of p-MLKL and ECs (n = 6 per group). **M,** CCK-8 results of cell viability of primary microvascular ECs (n = 4 independent experiments). **N-O,** Representative flow cytometry images and statistical analysis results of ECs stained with PI/annexin V. The necroptosis activation was represented by ratio of PI + /annexin V + (n = 5 per group). Data are mean ± SEM.

Based on these observations, we investigated whether DDIT4 knockdown alleviates BBB injury following ischemia-reperfusion. Immunofluorescence staining showed that DDIT4 expression increased markedly in BMECs after ischemia-reperfusion (Figure 5A), with a greater increase in aged mice (Figure 5C). To define the temporal dynamics of DDIT4 expression, we performed western blot analysis on ECs from aged mice at multiple time points following ischemia-reperfusion (Figure S8A). DDIT4 showed minimal co-localization with astrocytes (anti-GFAP) or microglia (anti-IBA1), indicating predominant expression in ECs (Figure S8B). We next assessed BBB disruption by immunofluorescence staining for fibrin in the ischemia-reperfusion cortex (Figure 5B). The extent of DDIT4 and CD31 co-localization was positively correlated with BBB permeability before and after ischemia-reperfusion. Aged mice showed a larger co-localization area and more extensive fibrin leakage than younger mice (Figure 5D).

*For in vivo endothelial specific knockdown*, Tie2-Cre mice aged to 20 months were used to drive Cre recombinase expression in ECs. We stereotactically injected AAVs carrying Cre-activated DDIT4 shRNA into the cortex (Figure 5E). Four weeks post-injection, ischemia was induced via MCAO followed by reperfusion according to the experimental workflow (Figure 5F). Western blotting and immunofluorescence confirmed efficient knockdown of DDIT4 in BMECs (Figure S9A-B). Consistent with these findings, immunofluorescence staining of fibrin deposition further demonstrated that DDIT4 knockdown protected BBB integrity (Figure 5G). Dual staining with TUNEL and PI showed reduced apoptosis and necrosis in BMECs from DDIT4 knockdown mice (Figure 5H, J, K). We then performed immunofluorescence staining for p-MLKL and CD31 in BMECs from the ischemia-reperfusion cortex (Figure 5I). Quantitative analysis showed that DDIT4 knockdown markedly reduced p-MLKL expression in BMECs (Figure 5L).

To mimic the pathological upregulation of DDIT4 observed in aged mice, we designed an AAV vector carrying endothelium-specific regulatory elements (Tie2-DDIT4) to induce DDIT4 overexpression in cortical microvascular ECs via stereotactic injection (Figure S10A-B). Western blotting and GFP fluorescence confirmed DDIT4 overexpression in targeted ECs (Figure S10C-D). Evans blue leakage assays showed that DDIT4 overexpression significantly worsened vascular permeability following ischemia-reperfusion injury (Figure S10E). In mice with endothelial specific DDIT4 overexpression, intraperitoneal Nec-1 administration after tMCAO reduced necroptosis related activation as reflected by lower p-RIP1/RIP1 and p-MLKL/MLKL (Figure S10F).

Simultaneously, primary BMECs were transfected with an adenovirus encoding DDIT4 to increase its endogenous expression. Consistent with the *in vivo* findings, CCK-8 assays shows that Nec-1, but not ZDEVD, improved the reduction in cell viability induced by DDIT4 overexpression (Figure 5M). Annexin V/PI flow cytometry further confirmed that Nec-1 decreases necroptosis in ECs induced by DDIT4 overexpression (Figure 5N-O). Conversely, DDIT4 knockdown reduced necroptosis in BMECs (Figure S11A-C). Together, these results indicate that DDIT4 knockdown in BMECs alleviates BBB disruption and suppresses necroptosis after stroke.

### DDIT4 exacerbates EC necroptosis by inducing ETC and mitochondrial dysfunction

**Figure 6:**
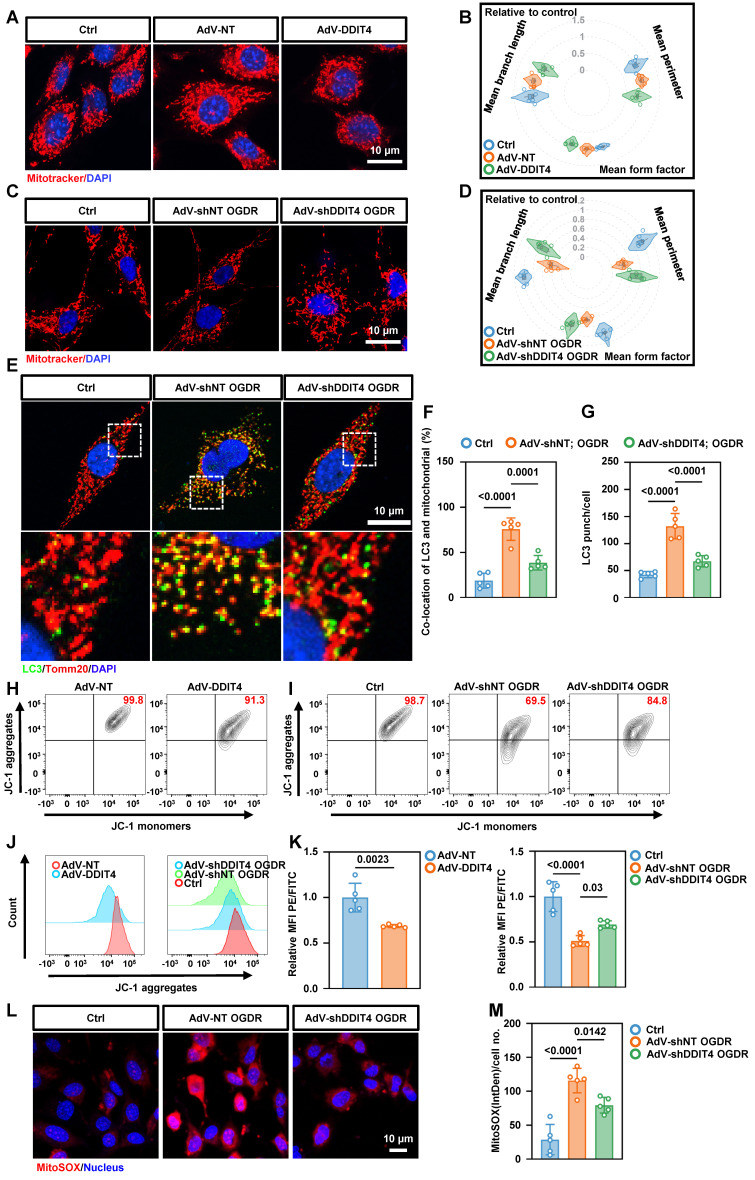
** Knockdown of DDIT4 in BMECs ameliorates mitochondrial dysfunction and reduces mtROS release. A,** Mitochondrial morphology in primary ECs with overexpression of DDIT4. **B,** Mitochondrial morphology in OGD/R-treated primary ECs after DDIT4 shRNA. **C-D,** Mean branch length (µm), mean perimeter (µm) and mean form factor (au) of mitochondria (n = 5 independent experiments). **E,** Representative images of LC3 co-stained with Tomm20 in primary BMECs after OGDR. **F-G,** The number of LC3 punch and co-location of LC3 and TOMM20 were presented (right). **H-I,** Mitochondrial membrane potential measured by flow cytometry of JC-1. JC-1 aggregation = normal mitochondrial membrane potential; JC-1 monomers = low membrane potential x-axis. **J,** Histograms display y-axis. **K,** Quantification of JC-1 flow cytometry (n = 5 per group). **L,** Representative images of staining with mitoSOX (red) and live cell nuclear stain hoechst33342 (blue). **M,** IntDen/cell measured using ImageJ (n = 5 per group). Data are mean ± SEM.

Mitochondrial instability is a well-established trigger of necroptosis^21^. Given the mitochondrial dysfunction observed in BMECs with abnormally elevated glycolysis, we further investigated the relationship between DDIT4 and mitochondrial function. Confocal imaging of primary BMECs revealed that DDIT4 overexpression reduced the number of linear mitochondria and increased punctate mitochondria, consistent with mitochondrial fragmentation (Figure 6A-B). Conversely, DDIT4 silencing attenuated mitochondrial fragmentation (Figure 6C-D) and OGDR-induced mitophagy (Figure 6E-G). Given that mitochondrial fragmentation can disrupt membrane potential, we assessed mitochondrial polarization using JC-1 dye, which forms red aggregates in healthy mitochondria and green monomers as potential declines. DDIT4 overexpression decreased the JC-1 red/green ratio, indicating mitochondrial depolarization, whereas DDIT4 knockdown reversed these changes following OGDR treatment (Figure 6H-K). To evaluate oxidative stress, we employed the mitochondrial-targeted superoxide indicator mitoSOX. OGDR increased mitoSOX signal in primary ECs, and this increase was attenuated by DDIT4 knockdown (Figure 6L-M). In addition, DDIT4-overexpressing BMECs displayed elevated autophagic activity (Figure S12A-F).

**Figure 7:**
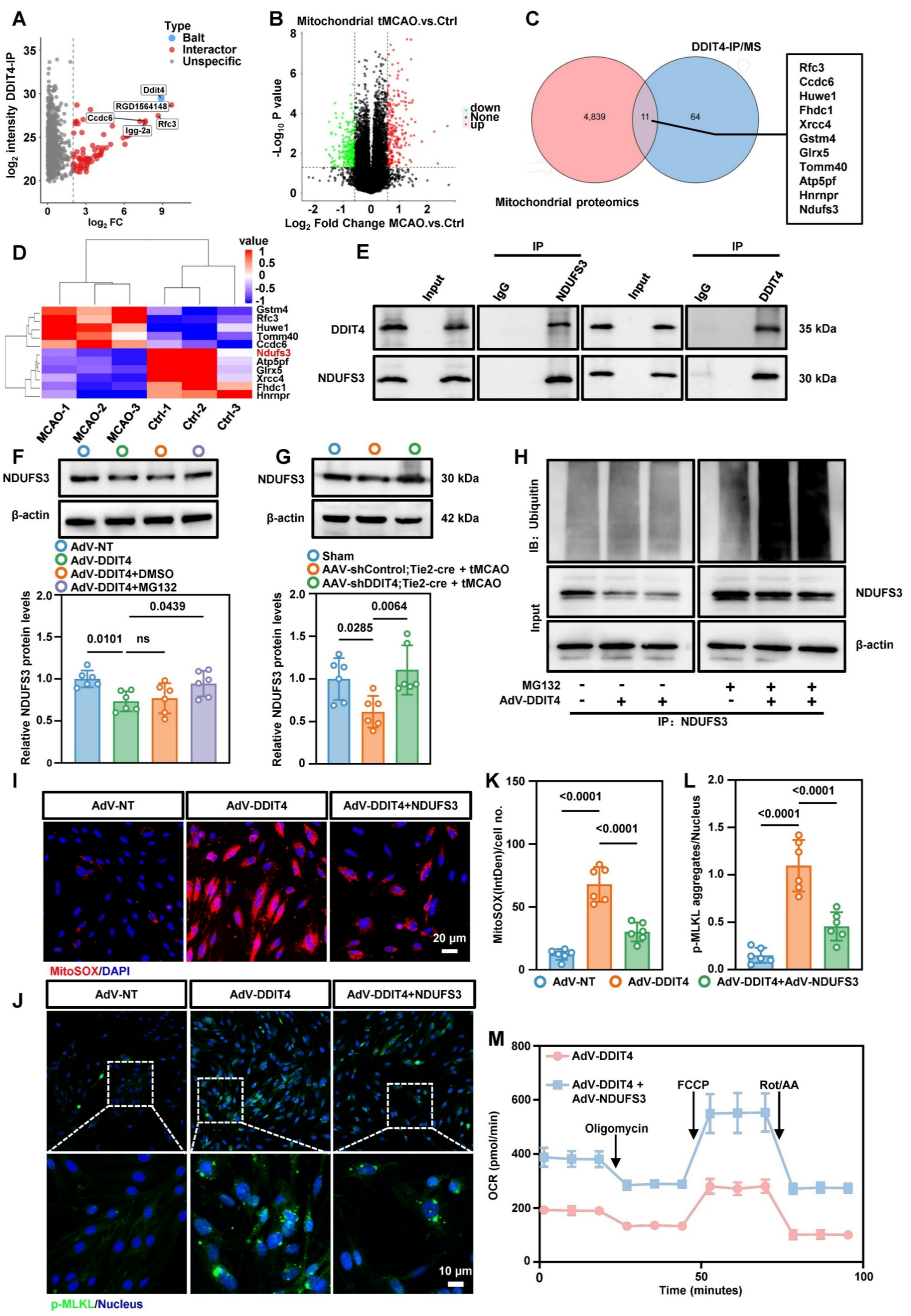
** DDIT4 facilitates the ubiquitination of NDUFS3 resulted in mitochondrial dysfunction. A,** Mass spectrometry analysis of proteins pulled down by anti-DDIT4 antibodies identified potential DDIT4-interacting proteins. **B,** Volcano plot of differentially expressed proteins between mitochondria isolated from cerebral microvascular tissues before and after ischemia-reperfusion. **C,** Overlapping analysis of differential proteins in mitochondrial proteomic and interacting proteins of DDIT4. **D,** Heat map showed the expression of 11 overlapping proteins in mitochondrial proteomic. **E,** Co-immunoprecipitation of NDUFS3 (left) or DDIT4 (right) in ECs (IgG as a control antibody). **F,** Western blotting of NDUFS3 in indicated cells. Endothelial cell was treated with MG132 6h for harvest. And the quantification of protein was shown in below (n = 6 per group). **G,** Western blotting of NDUFS3 in cerebral microvascular tissues from conditional DDIT4 knockdown mice. The quantification of protein was shown in below (n = 6 per group). **H,** Co-IP of NDUFS3 and then western blotted with anti-ubiquitin in DDIT4 overexpression ECs. **I,** Representative images of staining with mitoSOX (red) and live cell nuclear stain hoechst33342 (blue). **J,** Phospho-MLKL aggregation in indicated cells. **K,** IntDen/cell measured using ImageJ (n = 6 per group). **L,** p-MLKL aggregates/nucleus quantified using Fiji (n = 6 per group). **M,** The OCR was measured in indicated cells. Data are mean ± SEM.

To explore the mechanism underlying DDIT4-mediated mitochondrial dysfunction, we performed co-immunoprecipitation followed by mass spectrometry and identified 75 DDIT4-interacting proteins (Figure 7A). We then conducted a mitochondrial proteomics using mitochondria from cerebral microvascular tissues after tMCAO (Figure S13A). This analysis identified 4,850 mitochondrial proteins and revealed significant differences in expression before and after ischemia-reperfusion (Figure 7B). Integration of the mass spectrometry and mitochondrial proteomics datasets revealed 11 mitochondrial proteins as potential DDIT4 interactors (Figure 7C). Among these, NDUFS3, a core subunit of ETC complex I, was significantly reduced following ischemia-reperfusion (Figure 7D)^22^. Co-immunoprecipitation confirmed that DDIT4 directly binds NDUFS3, suggesting a regulatory role in its turnover (Figure 7E). To assess the involvement of protein degradation pathways, primary microvascular ECs were treated with MG132, a proteasome inhibitor, or chloroquine, a lysosomal acidification inhibitor. After DDIT4 overexpression, NDUFS3 levels decreased in DMSO treated control cells. This reduction was reversed by MG132 treatment (Figure 7F), but not by chloroquine (Figure S13B). Consistent with these findings, endothelial-specific knockdown of DDIT4 prevented the ischemia-reperfusion associated decrease in NDUFS3 levels (Figure 7G). We next investigated whether DDIT4 overexpression increases the ubiquitylation of endogenous NDUFS3. Co-IP assays for NDUFS3 and ubiquitin showed increased NDUFS3 ubiquitylation in MG132 treated ECs after DDIT4 overexpression (Figure 7H).

To further assess the functional role of NDUFS3, primary microvascular ECs were infected with adenoviruses expressing NDUFS3. Western blotting confirmed successful NDUFS3 overexpression (Figure S13C). NDUFS3 overexpression restored ETC complex I function (Figure S13D), reduced the release of mtDNA (Figure S13E) and mtROS (Figure 7I, K), and inhibited the formation of p-MLKL aggregates (Figure 7J, L). In addition, DDIT4 overexpression impaired basal respiration, maximal respiration, and ATP-linked respiration in primary microvascular ECs, and these defects were rescued by NDUFS3 overexpression (Figure 7M, Figure S13F-H). Collectively, these results demonstrate that DDIT4 induces ETC dysfunction and mitochondrial impairment, thereby promoting mtROS generation and necroptosis in BMECs.

### Glycolysis/H3K18la/ATF4-DDIT4 forms a pathological feedback loop in BMECs

**Figure 8:**
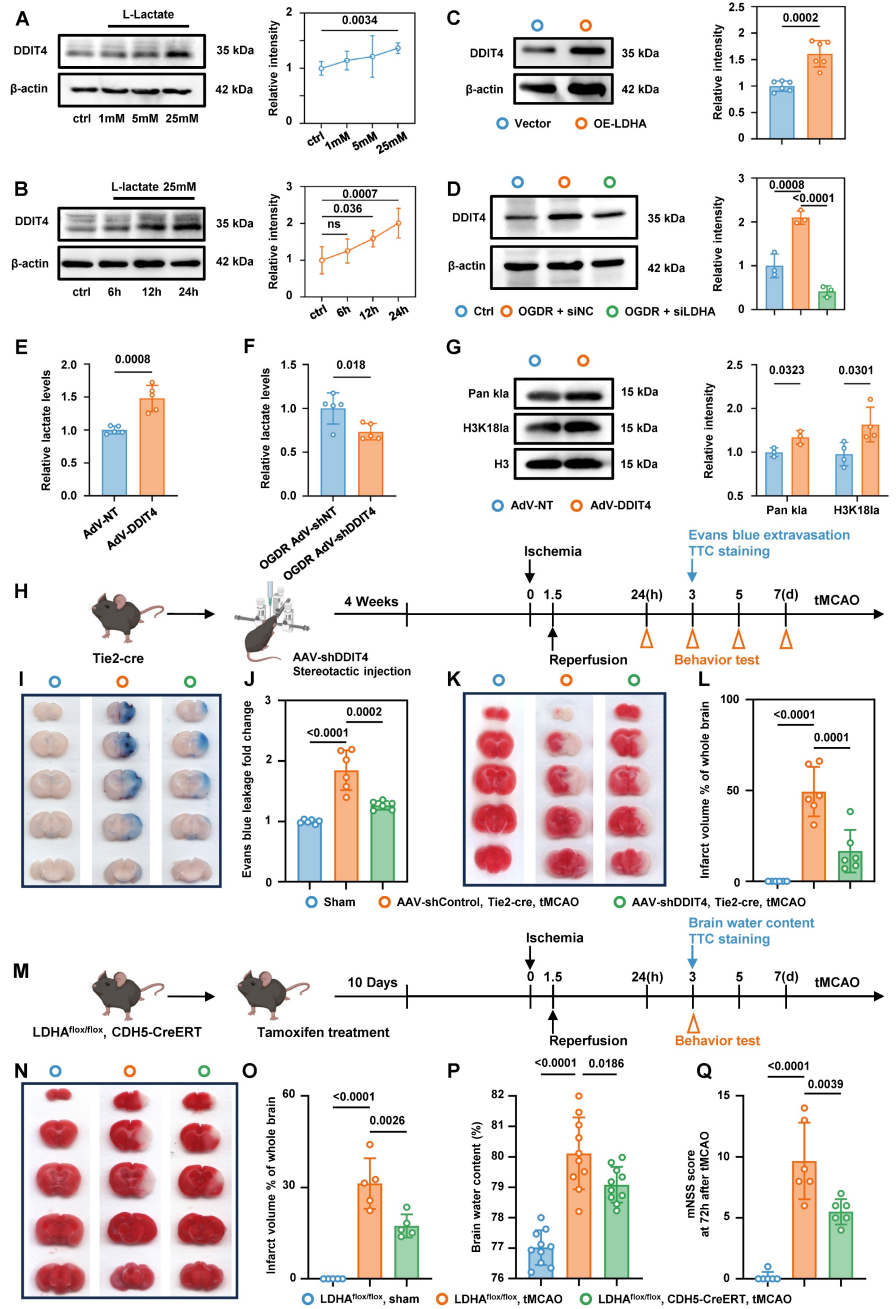
** Interruption of glycolysis/H3K18la/ATF4-DDIT4 feedback loop of BMECs alleviate edema and brain injury in tMCAO mice. A,** Western blotting of DDIT4 in primary BMECs treated with varying concentrations of lactate. The quantification of protein was shown in the right panel (n = 5 per group). **B,** Western blotting of DDIT4 in primary BMECs treated with lactate for different durations at the same concentration. The quantification of protein was shown in the right panel (n = 5 per group). **C,** Western blotting of DDIT4 in primary BMECs with or without LDHA overexpression. The quantification of protein was shown in the right panel (n = 6 per group). **D,** Western blotting of DDIT4 in OGDR treated primary BMECs with or without LDHA knockdown. The quantification of protein was shown in the right panel (n = 3 per group). **E,** Colorimetric assay for lactate levels in primary BMECs with or without DDIT4 overexpression (n = 5 per group). **F,** Colorimetric assay for lactate levels in OGDR treated primary BMECs with or without DDIT4 konckdown (n = 5 per group). **G,** Western blotting of Pan- and site-specific histone lactylation in primary BMECs with or without DDIT4 overexpression. The quantification of protein was shown in the right panel (n = 3-4 per group). **H,** Experimental design and timeline of ischemic stroke, DDIT4 knockdown, and analysis in tMCAO mice. **I-J,** Evans blue leakage of mice brains in coronal sections and extravasation (fold change relative to sham) from Sham, AAV-shControl Tie2-cre tMCAO, and AAV-shDDIT4 Tie2-cre tMCAO groups (n = 6 per group). **K-L,** Representative images and statistical results of TTC (n = 6 per group). **M,** Experimental design and timeline of ischemic stroke and analysis in LDHA^flox/flox^ mice and LDHA^flox/flox^; CDH5-CreERT mice. **N-O,** Representative images and statistical results of TTC (n = 5 per group). **P,** Statistical results of brain water content (n = 10 per group). **Q,** Modified neurological severity score at 3 days after tMCAO in different groups (n = 6 per group). Data are mean ± SEM.

Western blot analysis showed that exogenous lactate stimulation of primary microvascular ECs promoted DDIT4 expression in a dose- and time-dependent manner (Figure 8A-B). Plasmid-mediated overexpression of LDHA in primary microvascular ECs, which increased endogenous lactate production, also increased DDIT4 levels (Figure 8C). In contrast, siRNA-mediated knockdown of LDHA inhibited lactate production (Figure S14A) and reduced DDIT4 expression after OGDR treatment (Figure 8D). Notably, DDIT4 overexpression increased endogenous lactate production in primary microvascular ECs (Figure 8E) and was accompanied by a marked increase in Pan-Kla and H3K18la levels (Figure 8G). Conversely, DDIT4 knockdown reduced endogenous lactate production in ECs following OGDR treatment (Figure 8F). Together, these results support a pathological feedback loop - Glycolysis/H3K18la/ATF4-DDIT4 after ischemia-reperfusion.

### Interruption of glycolysis/H3K18la/ATF4-DDIT4 feedback loop in BMECs alleviate edema and brain injury in tMCAO mice

AAV-shDDIT4 was stereotactically injected into the brains of Tie2-Cre mice. Four weeks post-injection, ischemia was induced via MCAO, followed by a reperfusion phase, according to the experimental workflow (Figure 8H). Evans blue staining, TTC staining, and mNSS analysis indicated that suppressing DDIT4 in BMECs significantly attenuated edema and brain injury following ischemia-reperfusion (Figure 8I-L, Figure S14B).

We next generated mice with BMEC-specific LDHA deletion by crossing LDHA^flox/flox^ mice with CDH5-CreERT mice. To ablate LDHA in BMECs, 2-month-old mice were given 20 mg tamoxifen by intragastric administration for 5 consecutive days, after which experiments were performed according to the experimental workflow (Figure 8M). Consistent with the conditional DDIT4 knockdown, conditional knockout of LDHA in BMECs reduced edema and brain injury (Figure 8N-Q). qPCR analysis showed that disrupting this feedback loop reduced the expression of pro-inflammatory genes in the microvascular tissue (Figure S14C-D). Collectively, these data demonstrate that interruption of glycolysis/H3K18la/ ATF4-DDIT4 loop of BMECs alleviate edema and brain injury in tMCAO mice.

## Discussion

Ischemia-reperfusion induced microvascular endothelial barrier damage limits benefits of recanalization as cerebral edema and microvascular no-reflow correlate with clinical outcomes. Bioenergetic failure is an important determinant of cell fate^23^. However, the signals that initiate and sustain metabolic insufficiency of ECs, remain poorly understood. In this study, we found that higher serum lactate levels and L/P ratio were positively associated with cerebral edema and poorer neurological recovery in ischemic stroke patients following vascular recanalization, particularly in older individuals. In stroke mice, we identified a hyper-glycolytic endothelial subpopulation, characterized by mitochondrial dysfunction and activation of necroptosis, with a greater expansion in aged mic. This EC phenotype shift was driven by lactate-induced H3K18la, which epigenetically exacerbated metabolic insufficiency and ROS production through activation of the ATF4-DDIT4 axis. These findings identify a metabolic feedback loop glycolysis/H3K18la/ATF4-DDIT4 that promotes EC necroptosis and a pro-inflammatory microenvironment at the BBB, suggesting that targeting this axis may provide early warning marker and therapeutic strategy to mitigate ischemia-reperfusion injury.

Post-ischemia-reperfusion cerebral edema, particularly malignant edema, is a major contributor to early neurological deterioration and mortality after revascularization. This pathophysiological process not only undermines the benefits of recanalization but also predisposes patients to secondary neurological decline, ultimately influencing long-term prognosis^24^. In this study, we identified a distinct serum energy metabolite profile associated with poor outcomes. Elevated serum lactate and L/P ratios were associated with increased cerebral edema after recanalization, especially in elderly patients, supporting a direct link between bioenergetic disruption and edema formation. Cerebral edema and the no-reflow phenomenon are central drivers of ineffective reperfusion, often exacerbating each other^25^, ^26^. Analogous to myocardial infarction, where 35% of patients experience no-reflow due to reperfusion injury mediated by mitochondrial dysfunction^27^, the no-reflow phenomenon in stroke is dynamic and is often not detected by conventional imaging^2^. These observations highlight that targeting energy metabolism, in addition to restoring flow, may be critical for effective therapy.

Previously studies have shown that astrocyte-derived lactate supports neuronal activity under physiological and during early ischemia^28^. Early glycolytic activation in glial cells may be neuroprotective by supplying neurons with alternative energy substrates and delaying cell death. However, as ischemia progresses, excessive lactate production becomes detrimental, exacerbating neuronal injury and glial activation^29^. Cerebral edema after ischemia-reperfusion is largely driven by BBB disruption, with endothelial dysfunction playing a central role^24^. ECs exhibit the highest glycolytic activity among brain-resident cells, rendering them particularly susceptible to pathological lactate accumulation^15^. Our data show that modulating glycolytic flux in ECs reduces cell death and preserves BBB integrity, underscoring the importance of metabolic regulation and the interaction between age-associated metabolic shifts and ischemia-reperfusion injury.

Age-related immune and metabolic heterogeneity further amplifies stroke pathology^18^, ^29^. Although the neutrophil-to-lymphocyte ratio (NLR) remains a robust prognostic biomarker, interventions targeting neutrophils have not improved outcomes^30^. This may reflect the unappreciated contribution of metabolite-derived stress originating from neurovascular and immune cells. In senescent cells, mitochondrial dysfunction limits oxidative phosphorylation, forcing a reliance on glycolysis, which often fails to meet the heightened energy demands during ischemia^9^. Reperfusion introduces metabolic changes that are dynamic and spatially heterogeneous^31^. In aged individuals, hyperglycolysis combined with impaired mitochondrial bioenergetics may create a self-amplifying loop that promotes BBB disruption and exacerbates cerebral edema.

Functionally, experimental stroke models revealed that ECs adopt a transcriptional program enriched for glycolysis, mitophagy, and necrotic cell death pathways. Mitochondria also serve as platforms for necroptotic signaling and initiate cell death when their integrity is compromised^14^. Post-stroke, ECs exhibit elevated H3K18la and ATF4-DDIT4 signaling, which impairs mitochondrial function, enhances mitophagy, increases mitochondrial ROS (mtROS), and promotes RIPK3-MLKL-mediated necroptosis. Necroptosis not only mediates regulated inflammatory cell death but also amplifies local inflammation by activating proinflammatory gene expression. Lysine lactylation (Kla), a lactate-derived post-translational modification, has emerged as an important regulator in ischemia-reperfusion injury. For instance, H4K12la drives glycolysis-related gene expression in microglia, reinforcing inflammatory feedforward loops in AD^12^. Here, we identified H3K18la as a critical regulator of ATF4-DDIT4-mediated bioenergetic failure. The ATF4-DDIT4 axis, previously shown to promote glycolysis and autophagy in tumors^32^, similarly drives pathological glycolysis in ECs following stroke. The resulting glycolysis/H3K18la/ATF4-DDIT4 feedback loop triggers mitochondrial dysfunction, EC necroptosis, and BBB disruption. For patients with elevated lactate during reperfusion, early interventions targeting endothelial glycolysis or DDIT4 may mitigate this vicious cycle and improve outcomes.

In conclusion, our findings establish the glycolysis/H3K18la/ATF4-DDIT4 axis as a central mechanism driving post-ischemic cerebral edema and linking metabolic dysfunction to epigenetic regulation and BBB injury. From a translational perspective, we identify a biomarker signature comprising serum lactate, lactate-to-pyruvate ratio, and epigenetically regulated necroptotic factors for predicting adverse events and edema. These results provide mechanistic insight into metabolic control of EC fate in stroke and highlight potential early warning markers and therapeutic targets to prevent bioenergetic failure and associated edema. Further elucidating the "bioenergetic no-reflow" phenomenon and its governing epigenetic networks will be an important focus for future research.

## Materials and Methods

This study did not generate new unique reagents.

### Human serum samples

Serum samples for the detection of lactate and pyruvate were from Patients in the Multicenter Clinical Trial of Revascularization Treatment for Acute Ischemic Stroke (TRAIS), which retrospectively compiled data from all consecutive patients with AIS admitted to 14 stroke centers in China (ChiCTR2000033456). All participants provided informed consent, and the study adhered to the ethical standards outlined in the Declaration of Helsinki. The local institutional review committee approved all the research plans.

Cerebral edema was graded using the SITS-MOST system, where focal brain edema typically appears as a narrowing of cerebrospinal fluid spaces—like effacement of cortical sulci or ventricular compression. Based on MRI/CT scans performed within 24-72 hours post-stroke, patients were categorized into one of four grades: no cerebral edema (edema score = 0), edema affecting less than one-third of the hemisphere (edema score = 1), edema involving more than one-third of the hemisphere (edema score = 2), edema causing midline shift (edema score = 3). Serum samples were randomly selected according to prognosis and edema criteria.

Serum samples for metabolomic analysis were from 20 AIS patients who received intravenous thrombolysis at 4 comprehensive stroke centers in China, including Wuhan Union Hospital, People's Hospital of Dongxihu District, Hankou Hospital of Wuhan City and Hubei Provincial Hospital of Traditional Chinese Medicine and Western Medicine Integration. Similarly, the samples were randomly selected based on prognosis and edema criteria. The patient groups were as follows, those exhibiting a 90-day mRS Score of 3 or higher, an edema score of 1 or greater, or any evidence of intracranial hemorrhage were classified as having a poor prognosis. Conversely, patients with a 90-day mRS Score below 3, an edema score less than 1, and no signs of intracranial hemorrhage were categorized as having a good prognosis.

Comprehensive demographic and clinical information were presented in Table S1. The experimental protocol for the utilization of frozen serum samples received approval from the Ethics Committee of Union Hospital, Tongji Medical College, Huazhong University of Science and Technology. The serum samples were stored at -80 degrees Celsius and subsequently employed for further experimental analysis.

### Mice

All animal protocols were approved by the Medical Ethics Committee of Tongji Medical College and the Institutional Committee of Animal Care and Use, Huazhong University of Science and Technology (HUST), Wuhan, China.

CDH5-CreERT mice and LDHA^flox/flox^ mice were purchased from Shulaibao (Wuhan) Biotechnology Co., Ltd. To generate endothelia-specific LDHA-deficient mice, LDHA^flox/flox^ mice were crossed with CDH5-CreERT mice. Six-week-old male LDHA^flox/flox^; CDH5-CreERT mice and littermate LDHA^flox/flox^ mice underwent tamoxifen (Beyotime Biotechnology, China) treatment to induce Cre recombinase expression (intraperitoneal injection, 75 mg kg^-1^ for 5 consecutive days).

Tie2-cre mice (Cre expression driven by the Tie2 promoter) were obtained from Wuhan Shulaibao (Wuhan) Biotechnology Co., Ltd. All genotypes were confirmed through PCR amplification followed by agarose gel electrophoresis. The animals were housed in a controlled environment with constant humidity (40-60%) and temperature (20-25 °C), maintaining a 24-hour light-dark cycle. Mice had ad libitum access to water and food.

Young (8 weeks) and aged (18-24 months) adult male C57BL/6 mice were sourced from Wuhan Shulaibao (Wuhan) Biotechnology Co., Ltd. Five mice per cage were kept in a temperature-controlled room set at 21 °C, with humidity maintained between 40-60%, under continuous light-dark cycles lasting 24 hours.

### Establishment of tMCAO model

The method for establishing the mouse model is based on previously described protocols^33^. In summary, under 1.5% isoflurane anesthesia (administered at a flow rate of 0.8 L min^-1^), a paracentral median incision approximately 1 cm in length was made on the skin of the neck. Following blunt dissection to expose the submandibular gland, the right common carotid artery, external carotid artery, and internal carotid artery were identified. Subsequently, adipose tissue at the bifurcation of the common carotid artery was carefully removed. A monofilament measuring 4 millimeters in length with a silicone rubber-coated tip (diameter: 220 µm) (YUSHUN BIOTECH, Pingdingshan, China) was inserted into the external carotid artery and advanced along the right internal carotid artery until resistance was encountered. After occlusion for a duration of 60 minutes, reperfusion was achieved by withdrawing the fine filament. For sham-operated mice, arterial anatomical separation was performed without filament insertion. Throughout the procedure, mice were maintained at an optimal temperature using a heating pad set to 37 °C.

### Evans blue extravasation

Evans blue extravasation was performed for evaluating BBB integrity as previously described^34^. Briefly, mice were anesthetized 4 h after Evans blue (4% in saline solution, 2 mL/kg; Sigma Aldrich, St. Louis, MO, USA) intravenous administration, and then transcardially perfused with PBS. Randomly chosen mice continued to be perfused with 4% paraformaldehyde (PFA) and then those brains were cut into five sections each. The remaining brains were separated into left and right hemispheres respectively, weighed, homogenized with 1 mL of 50% formamide, and centrifuged (14,000 × g) for 30 min. Then, the supernatant was collected and mixed with absolute ethanol (1:3). Evans blue concentration (mg/g tissue) determined by absorbance at 630 nm with spectrophotometry represented BBB permeability.

### MRI study

MRI was performed at 1 days, 3 days and 5 days after tMCAO, respectively (7-T Bruker Biospec small animal MRI). The following three sequences were applied: T2, T1, and enhanced T1 as previously described. The isointense region on T2 images represented the normal brain tissue. Higher-intensity volume on T2-TSE images represented ischemic volume^35^. The total infarct volume was obtained by calculating the infarct volume at each section. Post-enhanced T1 images were acquired after intravenous gadolinium-diethylene triamine pentaacetic acid (Gd-DTPA) (0.2 mmol/kg^-1^, 20 min) injection. The product of the pre- and post-enhancement signal intensity change values on T1 imaging (T1SI-diff) and permeable BBB volume (PBV) reflected the extent of BBB leakage.

### Viral vectors

To specifically knock down ATF4 in BMECs *in vivo*, we constructed a central nervous system microvasculature EC-targeted AAV-BR1, which achieved gene silencing by utilizing shRNA that targets ATF4 transcripts within BMECs. AAV-shControl or AAV-shATF4 was administered via tail vein injection into mice 21 days prior to inducing cerebral ischemia/reperfusion injury (OBiO, Shanghai, China). To confirm successful transfection of the vector, mice injected with the AAV-mcherry vector virus were sacrificed 21 days post-injection. Brain frozen sections (20 μm) were prepared using a Leica CM1950 cryostat (Leica Microsystems GmbH, Germany). mCherry-positive cells in the cortex were visualized using confocal microscopy (Nikon, Japan).

To specific knockdown of DDIT4 in BMECs *in vivo*, an adenovirus containing double-floxed Cre-inducible mir155-shRNA targeted against DDIT4 was constructed (Genechem, Shanghai, China). Adeno-associated virus targeting DDIT4 was administered into the lateral ventricles of mice 21 days prior to inducing cerebral ischemia/reperfusion injury. Briefly, the mice were secured in a stereotactic frame under 1.5% isoflurane anesthesia (delivered at a rate of 0.8 L/min). A microsyringe with a capacity of 2 μL was utilized to inject an adeno-associated virus (AAV) suspension at a concentration of 1 × 10^12^ TU/mL, at a flow rate of 0.2 μL/min (total volume: 1 μL). The coordinates for the injection sites were as follows: AP, -1 mm; ML, -1 mm; DV, -1.5 mm. Following the injection, it was essential to maintain the syringe in its original position for no less than 10 minutes before gradually withdrawing it to prevent backflow and ensure thorough dispersion of the virus. Further, after a period of 21 days post-injection of the vector virus, the mice were euthanized for preparation of frozen sections (20 μm). The expression levels of DDIT4 within the injection area were subsequently examined using a confocal microscope (Nikon, Japan).

To specifically overexpress DDIT4 in BMECs, AAV-DDIT4 or AAV-GFP was injected into the lateral ventricles of mice 21 days prior to inducing cerebral ischemia/reperfusion injury (Genechem, Shanghai, China). Briefly, the mice were anesthetized with 10% pentobarbital and secured onto a stereotactic frame. Using a 2 μL microsyringe, an adeno-associated virus (AAV) suspension at a concentration of 1 × 10^12^ TU/mL was injected at a rate of 0.2 μL/min for a total volume of 1 μL. The coordinates for the injection sites were as follows: anterior-posterior (AP), -1 mm; medial-lateral (ML), -1 mm; dorsal-ventral (DV), -1.5 mm. After the injection, it was essential to keep the syringe in its original position for at least 10 minutes before slowly withdrawing it to prevent backflow and ensure complete dispersion of the virus. The AAV-GFP group received an equivalent volume of vector virus administered at the same site. To confirm the successful transfection of the vector, mice were sacrificed 21 days post-injection of the AAV-GFP vector. Brain frozen sections (20 μm) were prepared using a Leica CM1950 cryogenic microtome (Leica Microsystems GmbH, Germany). GFP-positive cells in the peri-ischemic area were visualized using confocal microscopy (Nikon, Japan).

### PI/TUNEL staining

PI/TUNEL staining was performed as previously reported^36^. Briefly, PI was intraperitoneally injected (1 μg/g; Sigma-Aldrich, St. Louis, MO, USA) while the tMCAO model was established. Cell apoptosis was detected via TUNEL (Beyotime, China) staining. Frozen brain sections were incubated with TUNEL (37 °C for 1 h), washed three times with PBST (PBS with 0.5 ‰ Tween-20), and then mounted and visualized with a Nikon A1-Si confocal microscope (Nikon, Japan). As reported previously, PI-/TUNEL+ and PI+/TUNEL+ indicate apoptosis and necrosis, respectively.

### Primary culture of mouse BMEC

The six-well plate was coated overnight with the adhesion factor (AF). Mouse BMECs were isolated from C57BL/6 mice, following a modified protocol^36^. The mouse cerebral cortex was minced into small fragments and digested in DMEM (Hyclone) supplemented with 100 µL/mL DNase I and 0.1% collagenase IV at 37 °C for 60 minutes. Subsequently, the mixture was centrifuged at 500 × g at 4 °C for 5 minutes.

The resulting pellet was resuspended in DMEM containing 17% Percoll (Amersham Pharmacia Biotech, Piscataway, New Jersey, USA) and subjected to centrifugation at 2500 × g at 4 °C for an additional 10 minutes. The sediment collected at the bottom of the tube was then suspended in DMEM containing 33% Percoll and further centrifuged under the same conditions for gradient separation. Purified microvessels were harvested from the middle layer of suspended material and washed twice with cold PBS. Following this purification process, the microvessels were inoculated into six-well plates containing endothelial cell medium (#1001, ScienCell Research Laboratories, San Diego, California, USA) and incubated at 37 °C in a humidified atmosphere with 5% CO2. After three days, ECs were plated in six-well plates (1-1.5 × 10^6^ per plate) or 12-well plates (3-4 × 10^5^ per plate) with fresh medium.

### Chromatin immunoprecipitation (ChIP)

The cells were inoculated in 10 cm culture dishes and incubated overnight, followed by treatment with a 37% formaldehyde solution^37^. Chromatin immunoprecipitation (ChIP) analysis was conducted using the SimpleChIP Enzymatic Chromatin Immunoprecipitation Kit (#9003, Cell Signaling Technology), adhering to the manufacturer's instructions. The antibodies employed included anti-ATF4, anti-H3K18la, and control rabbit IgG. Table S4 provides a list of the qRT-PCR primers utilized for DNA precipitation.

### Immunofluorescent staining

Immunofluorescence staining was conducted as previously described^12^. All mice underwent cardiac perfusion with phosphate-buffered saline (PBS), followed by fixation of brain tissue in 4% paraformaldehyde. Subsequent to gradient dehydration using a sucrose solution, the samples were embedded in OCT compound, and the frozen blocks were sectioned into slices measuring 10-20 μm. The following primary antibody was utilized: p-MLKL (Rb, 1:50, ab196436; Abcam, UK), Iba1 (Goat, 1:50, ab5076; Abcam, UK), GFAP (Ms, 1:50, 60190-1-Ig; proteintech, China), CD31 (Goat, 1:50, AF3628; RD systems, USA), Fibrinogen (Sheep, 1:50, ab118533; Abcam, UK), DDIT4 (Rb, 1:50, ab191871; Abcam, UK), GLUT1 (Rb, 1:50, ab115730; Abcam, UK), Collagen IV (Goat, 1340-01, 1:50, SouthernBiotech), Lc3 (Rb, 1:50, ab192890; Abcam, UK), Tomm20 (Mouse, 1:50, ab56783; Abcam, UK), Lectin (1:50, DL-1177-1, VectorLaboratories). The secondary antibody is detailed in Table S2. Cell nuclei were stained with 4',6-diamidino-2-phenylindole (DAPI, Invitrogen). The samples were visualized using a Nikon A1Si confocal microscope (Nikon, Japan). Co-localization and the percentage of positive area images were analyzed using NIS Elements AR Imaging Software version 4.10 (Nikon) and ImageJ version 1.41 software.

### Histone extraction

According to the aforementioned report, an acid extraction method was employed to isolate histones from tissues or cells, with minor modifications made to the original protocol^38^. In summary, tissues or cells were collected and resuspended in lysis buffers containing protease inhibitors (10 mM Tris-HCl, 1 mM KCl, 1.5 mM MgCl2, and 1 mM DTT at pH 8.0) to facilitate the extraction of cell nuclei. Subsequently, the isolated cell nuclei were resuspended in an acidic solution (0.2 M H2SO4), and histones were extracted overnight at 4 °C. The mixture was then centrifuged at 16,000 g for 10 minutes at 4 °C. The supernatant was collected and histones were precipitated on ice using trichloroacetic acid to a final concentration of 35%. After washing and drying the precipitate, it was dissolved in water in preparation for Western blotting analysis.

### Western blotting analysis

Western blotting analysis was conducted following the previously established protocol 39. Equal amounts of protein samples (20-50 µg) were utilized for detection. Brain tissue or cells were lysed using RIPA lysis buffer supplemented with a mixture of protease and phosphatase inhibitors. The protein concentration of the resulting lysate was quantified using the BCA method and adjusted to ensure uniform final concentrations across samples. Following a 10-minute incubation in a water bath at 100 °C, equal quantities of proteins from the lysate were separated by SDS-PAGE and subsequently transferred onto a polyvinylidene fluoride (PVDF, Millipore) membrane. At room temperature, the membrane was blocked for 1 hour with a solution containing 5% skimmed milk prepared in Tris-buffered saline with 0.1% Tween-20 (TBST). It was then incubated overnight at 4 °C with the specified primary antibody. After three washes with TBST, the membrane was treated with a secondary antibody conjugated to horseradish peroxidase (HRP). Finally, immunoreactive proteins were detected using enhanced chemiluminescence (ECL) substrates and visualized on autoradiography film (Kodak). The following primary antibody was utilized: Pan Kla (PTM-1401, PTM BIO), H3K9la (PTM-1419, PTM BIO), H3K14la (PTM-1414, PTM BIO), H3K18la (PTM-1406, PTM BIO), H4K5la (PTM-1407, PTM BIO), H4K8la (PTM-1415, PTM BIO), H4K12la (PTM-1411, PTM BIO), H4K16la (PTM-1417, PTM BIO), H3 (PTM-1001, PTM BIO), H4 (PTM-1015, PTM BIO), LDHA (19987-1-AP, Proteintech), β-actin (Abclonal), DDIT4 (10638-1-AP, Proteintech), p-MLKL (ab196436, Abcam), MLKL (37705, Cell Signaling Technology), p-RIP1 (65746S, Cell Signaling Technology), RIP1 (7519-1-AP, Proteintech), p-mTOR (5536S, Cell Signaling Technology), mTOR (66888-1-Ig, Proteintech), NDUFS3 (15066-1-AP, Proteintech), Ubiquitin (43124S, Cell Signaling Technology), ATF4 (60035-1-Ig, Proteintech). The intensity of the band was quantified using ImageJ software.

### Processing of scRNAseq data

We obtained the mice 2 days after tMCAO or sham surgery datasets from publicly available repositories. Including young mice and aged mice. Raw data were downloaded from GSE225948. Seurat pipeline Clustering method is Seurat 4.0(R package). Cells whose gene number was less than 200, or gene number ranked in the top 1%, or mitochondrial gene ratio was more than 25% were regarded as abnormal and filtered out. Dimensionality reduction was performed using PCA, and visualization was realized by TSNE and UMAP.

### Targeted energy metabolomics analysis

The serum from patients undergoing endovascular recanalization treatment is preserved at a temperature of -80 degrees Celsius. Subsequently, the serum samples were dispatched to Cosmos Wisdom in Hangzhou, China, for analysis via liquid chromatography-mass spectrometry (LC-MS). Frozen samples were thawed and vortexed for 10 s to ensure homogeneity. An aliquot of 50 µL was transferred into a pre-labeled 1.5 mL centrifuge tube, followed by the addition of 250 µL of 20% acetonitrile/methanol extraction solution. After vortexing for 3 min, the mixture was centrifuged at 12,000 r/min for 10 min at 4 °C. A 250 µL aliquot of the resulting supernatant was transferred to another labeled tube and allowed to precipitate proteins at -20 °C for 30 min. The samples were then centrifuged again at 12,000 r/min for 10 min at 4 °C. Subsequently, 180 µL of the clarified supernatant was passed through a protein precipitation plate and used for instrumental analysis; the prepared injection solutions were stored at -20 °C until analysis. Metabolite profiling was performed using an Ultra-Performance Liquid Chromatography (UPLC) system (Waters ACQUITY H-Class) coupled to a triple quadrupole tandem mass spectrometer (QTRAP® 6500+). A Waters ACQUITY UPLC BEH Amide column (1.7 µm, 100 × 2.1 mm i.d.) was employed. Electrospray ionization (ESI) was performed at 550 °C with ion spray voltages of +5500 V in positive mode and -4500 V in negative mode. The curtain gas was set to 35 psi. For each analyte, optimized declustering potentials (DP) and collision energies (CE) were applied during Multiple Reaction Monitoring (MRM) acquisition on the QTRAP 6500+. Qualitative metabolite identification was achieved by matching detected ions to an in-house database constructed from authenticated reference standards. Quantification was performed using Multiple Reaction Monitoring (MRM) on the triple quadrupole system. In MRM mode, the first quadrupole isolates the precursor ion of the target analyte while excluding ions of other m/z values. After collision-induced dissociation in the collision cell, specific product ions are filtered by the third quadrupole, minimizing interference and improving precision and reproducibility. Chromatographic peak areas of each target metabolite across samples were integrated and quantified using calibration curves generated from corresponding standards.

### CUT&Tag

The CUT&Tag experiments of microvascular tissues were completed by Wuhan GeneRead Biotechnology Co., Ltd. The extracted nuclei (~100,000 per reaction) were gently resuspended in 50 µL antibody buffer containing the primary antibody (H3K18la, #PTM-1427, active motif). Following an overnight incubation at 4 °C, the primary antibody was removed via centrifugation and the nuclei were incubated in 50 µL wash buffer with a secondary antibody (IgG, proteintech, B900210) for approximately 2 h at 4 °C. Nucleus were washed using the magnet stand twice for 1 min in 500 μL Dig-Wash buffer to remove unbound antibodies. A 1:200 dilution of pG-Tn5 adapter complex (~0.04 μM) was prepared in Dig-300 Buffer (0.01% Digitonin, 20mM HEPES, pH 7.5, 300mM NaCl, 0.5mM Spermidine, 1× Protease inhibitor cocktail). After removing the liquid, 100 μL was added to the cells with gentle vortexing, which was incubated with pG-Tn5 at room temperature (RT) for 1 h. Nucleus were washed twice for 1 min in 500 μL Dig-300 Buffer to remove unbound pG-Tn5 protein. Next, nucleus were resuspended in 300 μL Tagmentation buffer (10mM MgCl2 in Dig 300 Buffer) and incubated at 37 °C for 1 h. To stop the tagmentation reaction, 10 µL 0.5 M EDTA, 3 µL 10% SDS and 2.5 µL 20 mg mL^-1^ protease K were added, and the mixture was incubated for 1 h at 50 °C. The DNA was subsequently extracted using phenol to chloroform to isoamyl alcohol, precipitated with ethanol and resuspended in double-distilled water. The DNA was amplified by PCR reaction as follows: 3 min at 72 °C and 30 s at 98 °C followed by 16 cycles of 15 s at 98 °C and 30 s at 60 °C and 30 s at 72 °C, with a final extension at 72 °C for 3 min. Finally, the amplified DNA was purified using Ampure XP beads (Beckman Counter). The final library was sequenced on the Illumina NovaSeq X Plus platform (San Diego, CA, United States) in PE150 mode.

### Statistical analysis

All data are expressed as mean ± standard error of the mean (SEM) unless otherwise described. Statistical analyses were performed using GraphPad Prism software (version 8.0) and the significance of differences was assessed by two tail unpaired Student's t test or one-way or two-way analysis of variance (ANOVA) followed by Tukey's multiple comparisons test. Correlation analysis was performed using the Pearson correlation test. The statistical parameters can be found in the figures and figure legends.

## Supplementary Material

Supplementary methods, figures and tables.

## Figures and Tables

**Table 1:** Results of univariate analysis of 90-day mRS

Factor	90 mRS 3-6	90 mRS 0-2	OR (95% CI)	Pvalue
Lactate				
> 2.2µmol/mL	57	62	2.247 (1.166-4.33)	0.014
≤ 2.2µmol/mL	18	44		
L/P				
> 10	58	67	1.986 (1.017-3.879)	0.043
≤ 10	17	39		

**Table 2:** Results of univariate analysis of 90-day mRS stratified by age

Factor	90 mRS 3-6	90 mRS 0-2	OR (95% CI)	Pvalue
Age ≥ 75				
Lac > 2.2µmol/mL	25	15	4.444(1.427-13.839)	0.008
Lac ≤ 2.2µmol/mL	6	16		
L/P > 10	24	17	2.824(0.94-8.479)	0.06
L/P ≤ 10	7	14		
55 ≤ age < 75				
Lac > 2.2µmol/mL	22	23	2.551(0.844-7.704)	0.092
Lac ≤ 2.2µmol/mL	6	16		
L/P > 10	24	27	2.667(0.758-9.383)	0.119
L/P ≤ 10	4	12		
age ≤ 54				
Lac > 2.2µmol/mL	10	24	0.833(0.244-2.841)	0.771
Lac ≤ 2.2µmol/mL	6	12		
L/P > 10	10	23	0.942(0.278-3.189)	0.924
L/P ≤ 10	6	13		

**Table 3:** Results of univariate analysis of edema score

Factor	Edema score 1-3	Edema score 0	OR (95% CI)	P value
Lactate				
> 2.2µmol/mL	62	57	2.659(1.38-5.123)	0.003
≤ 2.2µmol/mL	18	44		
L/P				
> 10	62	63	2.078(1.072-4.025)	0.029
≤ 10	18	38		

**Table 4:** Results of univariate analysis of edema score stratified by age

Factor	Edema score 1-3	Edema score 0	OR (95% CI)	P value
Age ≥ 75				
Lac > 2.2µmol/mL	25	15	3.571 (1.186-10.752)	0.021
Lac ≤ 2.2µmol/mL	7	15		
L/P > 10	27	14	6.171 (1.87-20.362)	0.002
L/P ≤ 10	5	16		
55 ≤ age < 75				
Lac > 2.2µmol/mL	24	21	2.449 (0.839-7.15)	0.097
Lac ≤ 2.2µmol/mL	7	15		
L/P > 10	22	29	0.59 (0.19-1.831)	0.359
L/P ≤ 10	9	7		
age ≤ 54				
Lac > 2.2µmol/mL	13	21	2.167 (0.585-8.021)	0.242
Lac ≤ 2.2µmol/mL	4	14		
L/P > 10	13	20	2.438 (0.661-8.992)	0.175
L/P ≤ 10	4	15		

## Abbreviations

ECs: endothelial cells
ROS: reactive oxygen species
EndMT: endothelial-to-mesenchymal transition
PCA: principal component analysis
L/P: lactate-to-pyruvate
2-DG: 2-deoxy-D-glucose
tMCAO: transient middle cerebral artery occlusion
scRNA-seq: single-cell RNA sequencing
GO: Gene Ontology
ECAR: extracellular acidification rate
OGDR: oxygen-glucose deprivation and reperfusion
OCR: oxygen consumption rate
qChIP: quantitative chromatin immunoprecipitation
TTC: 2,3,5-triphenyltetrazolium chloride
TEER: trans-endothelial electrical resistance
PI: propidium iodide
