# Supplementary material for: Microvascular endothelial metabolic dysfunction drives cerebral edema through bioenergetic failure after ischemia-reperfusion
Source: Theranostics. 2026 Jan 1;16(7):3577–98. doi: 10.7150/thno.127083 (PMC12846758; doi:10.7150/thno.127083)
Supplement: Supplementary file 1 — Supplementary methods, figures and tables. [file thnov16p3577s1.pdf]

Supplementary Materials for

Microvascular endothelial metabolic dysfunction drives cerebral edema through bioenergetic failure after ischemia-reperfusion

Yi-Fan Zhou[1#], Si-Bo Yang[1#], Feng Zhang[1#], Xiao-Di Sun[1], Bo-Hao Chang[1], Ya-Nan Li[1], Jie-Hong Wu[1], Hui-Juan Jin[1], Ming Huang[2], Sheng-Cai Chen[1], Hang Yang[1], Dong-Ya Zhu[3], Bijoy K Menon[4] and Bo Hu[1]*

Yi-Fan Zhou, Si-Bo Yang and Feng Zhang contributed equally to this work

[1]Department of Neurology, Union Hospital, Tongji Medical College, Huazhong University of Science and Technology, Wuhan 430022, China.

[2]Department of Neurology, Hubei Provincial Hospital of Integrated Chinese and Western Medicine, Hubei University of Chinese Medicine, Wuhan, China.

[3]Department of Clinic Pharmacology, School of Pharmacy, Nanjing Medical University, Nanjing 211166, China.

[4]Department of Community Health Sciences, Department of Clinical Neurosciences, Department of Radiology, and Hotchkiss Brain Institute, University of Calgary, AB, Canada.

***Corresponding author:**

Bo Hu

Department of Neurology, Union Hospital, Tongji Medical College, Huazhong University of Science and Technology, Wuhan 430022, China.

Tel: +86 27 85726028

E-mail: hubo@mail.hust.edu.cn

The file includes:

Materials and Methods

Fig S1 to S13

Table S1 to S6

MATERIALS AND METHODS

Behavioral analyses

The mNSS grades neurologic function on a scale of 0 to 14 (normal score 0; maximal deficit score 14). mNSS is a composite of motor and balance tests. Behavioral tests were performed by a researcher blinded to the treatment group during the light phase of the circadian cycle beginning 4h after lights were turned on.

2,3,5-triphenyltetrazolium chloride staining

Mice were euthanized and their brains were collected after 72h of reperfusion for 2,3,5-triphenyltetrazolium chloride (TTC) histology (G1017; Servicebio, Wuhan, China). The animals were euthanized and their brains were extracted and immediately frozen at -20°C for 20 min. During this time, TTC (1% in phosphate-buffered saline) was freshly prepared. The brains were sectioned coronally to 1-mm-thick slices and stained with 1% TTC at 37°C for 30 min. The slices were turned over every 15 min. Then, the brain slices were placed in 4% paraformaldehyde (PFA) for 1 h. Normal brain tissue stained brightly, and the infarcted areas were pale white.

Brain microvessel (MV) isolation

For each preparation, mice were anesthetized with 5% isoflurane, after which their brains were harvested and placed in DMEM high glucose medium at 4 °C. The cerebellum, meninges, choroid plexus, brainstem, and large superficial blood vessels were carefully removed. Subsequently, the cortical tissues were isolated and collected

separately. The cortical tissue was then cut into small pieces within the DMEM high glucose medium and homogenized using a glass homogenizer. The resulting homogenate was centrifuged (2000 g for 5 minutes at 4 °C) to eliminate the DMEM high glucose medium. The pellet was resuspended in a solution of 15% dextran (Sigma/Aldrich, St. Louis, Missouri, USA) and subjected to a second round of centrifugation (4000 g for 30 minutes at 4 °C) to isolate microvessels. The microvascular precipitate was resuspended in DPBS containing 1% BSA; thereafter, the supernatant underwent further centrifugation (4000 g for 10 minutes at 4 °C) to obtain the final microvascular tissue.

Isolation of ECs from adult mouse brains

Indicated-age adult mice were deeply anesthetized with pentobarbital sodium and perfused transcardially with ice-cold saline. Whole brain without cerebellum was immediately removed and washed with prechilled phosphate-buffered saline (PBS). The brain tissue was mechanically and enzymatically dissociated using the Adult Brain Dissociation Kit (130-107-677, Miltenyi Biotec) according to the manufacture's instruction. After myelin was removed by using the Cell Debris removal buffers, total cell pellet was resuspended with PBS containing 0.5% BSA followed by specific cell type isolation. Briefly, ECs were isolated by anti-CD31-coated MicroBeads (130-097-418, Miltenyi Biotec) by using a MACS multistand separator according to the manufacture's instruction.

Oxygen-Glucose Deprivation (OGD) followed by Re-oxygenation (OGD/R)

Cells were cultured with glucose-free medium in a hypoxic chamber (Thermo Fisher Scientific, USA, 1% O2, 94% N2, and 5% CO2) at 37 °C for 6 h. Then cells were returned to normal cell culture incubators (95% air and 5% CO2) with ECM (#1001, ScienCell Research Laboratories, San Diego, California, USA) for 2–12 h. Control cells were incubated in ECM under normal culture conditions for the same time period.

qPCR

Total RNA was extracted from tissues or cells using Trizol reagent (Vazyme). Subsequently, the samples were reverse transcribed utilizing a cDNA Synthesis Kit (Vazyme). Following this, quantitative PCR (qPCR) based on SYBR-Green was conducted to assess gene expression levels. The primer sequences used for qPCR are provided in Table S3. mRNA expression levels were standardized against the expression level of β-actin.

Measurement of lactate levels

The lactate content in microvessels or primary endothelial cells was assessed using the lactate content detection kit (BC2235, Solarbio). Tissue samples were homogenized in an ice bath, while cell extracts underwent ultrasonic treatment for 3 minutes. The samples were then centrifuged at 12,000 g for 10 minutes to collect the supernatant. Absorbance values of both tissue and cell supernatants, along with a series of standard

solutions, were measured at 570 nm. The absorbance values are directly proportional to lactic acid production. The concentration of lactic acid in the supernatant was quantitatively determined using a standard curve.

Mitochondrial morphology

Mitochondria in primary endothelial cells were labeled with MitoTracker Red (ThermoFisher, 100 nmol/L) at 37 °C for a duration of 30 minutes. Subsequently, they were observed using an inverted single-photon laser confocal scanning microscope (Zeiss, LSM780, Germany). Utilizing a 63× 1.4 NA oil-immersed objective lens, the mitochondrial fluorophores were excited with either a 561 nm or a 559 nm laser, and fluorescence emissions within the range of 570-616 nm were recorded. The length of the mitochondria was determined and quantified using ImageJ software.

Plate based assays for cell viability and reactive oxygen species (ROS)

Primary brain microvascular endothelial cells were inoculated into 96-well plates at a density of 2 × 10^4 cells per well. Adenovirus-mediated overexpression or knockout of DDIT4 was performed two days prior to the assessment of cell viability. The inhibitors Nec-1 (10 mM) and ZDEVD (25 μM) were mixed and incubated with the respective groups of cells for 24 hours in advance. On the day of detection, cell viability was evaluated using CCK-8 detection reagent (Vazyme, China). A total of 10 μL of CCK-8 solution was added to each well, and the cells were incubated in an incubator

maintained at 37 °C with 5% CO2. Absorbance readings at 450 nm were taken at time points: 0 h, 2 h, 4 h, 6 h, and 8 h. The percentage of cell survival was calculated based on absorbance values from the experimental group compared to those from both control and blank groups.

For reactive oxygen species (ROS) determination, mitoSOX (ThermoFisher) was utilized to detect mitochondrial superoxides. Following the manufacturer's instructions, a stock solution of MSR at a concentration of 5 mM was prepared using DMSO; subsequently, a working solution containing a concentration of 500 nM was made using HBSS supplemented with calcium and magnesium. Cells were stained with this working solution in an incubator set at 37 °C with an atmosphere containing 5% CO2 for a duration of thirty minutes. After staining, cells were washed with preheated HBSS; then average fluorescence intensity was analyzed utilizing NIS Elements AR Imaging Software version 4.10 (Nikon) alongside ImageJ version 1.41 software.

Seahorse metabolic assays

The Seahorse XF Mitochondrial Stress Test Kit was prepared in accordance with Agilent's established protocol, and subsequent analysis was conducted using WAVE software on the Agilent Seahorse XF 24-well analyzer. One day prior to the test, primary brain microvascular endothelial cells were inoculated overnight at a density of 4 x 10^4 cells per well. On the day of testing, the cells were processed following the manufacturer's instructions and analyzed utilizing the Agilent Seahorse Mitochondrial Stress Test Kit (Agilent).

Flow cytometry

Cell death was assessed using flow cytometry in conjunction with the Annexin V-FITC apoptosis detection Kit (Beyotime, C1062). Briefly, primary brain microvascular endothelial cells from various groups were trypsinized with 0.25% trypsin (without EDTA), and 1 × 10^6 cells were counted and washed twice with cold PBS. Following centrifugation at 2500 rpm for 5 minutes, the cells were resuspended in 200 μL of binding buffer. After sequential staining with 5 μL of FITC-conjugated annexin V and 10 μL of propidium iodide (PI), the cells were analyzed using a flow cytometer. Data analysis was performed utilizing FlowJo software.

Mitochondrial membrane potential

The mitochondrial membrane potential was assessed using the JC-1 kit (C2003S, Beyotime). Briefly, after transfecting primary brain microvascular endothelial cells with the appropriate adenovirus or incubating them with the corresponding inhibitor, the cells were detached from the culture plate using trypsin to create a cell suspension. Subsequently, they were incubated with JC-1 (2 μM) at 37 °C for 20 minutes. Following this incubation, the cells were washed twice with PBS containing 4% FBS in preparation for flow cytometry analysis. Post-acquisition data analysis was performed using Flow-Jo software. Aggregates were quantified under Texas Red (610/20, 600LP), while monomers were measured under FITC (525/50 505LP).

Trans-endothelial electrical resistance and dextran permeability

First, the Tranwell chamber (a polycarbonate membrane with a diameter of 6.5 mm and a pore size of 0.4 microns; Corning, New York State, USA) was incubated overnight at 37 °C in the presence of an adhesion factor. Endothelial cells were inoculated into the small chamber and cultured for three days to establish an endothelial cell barrier. The transendothelial electrical resistance (TEER) values were measured using an epithelial volt/ohmmeter equipped with STX2 electrodes (World Precision Instruments, Sarasota, Florida, USA).

Additionally, FITC-conjugated glucan (Mw 10,000) was utilized to assess permeability as previously described by Sigma-Aldrich. Briefly, 300 μg/mL of FITC-glucan was added to the upper chamber while 500 μL of culture medium devoid of FITC-glucan was introduced into the lower chamber. At intervals of 15-, 30-, 60-, and 120-minutes post-addition of FITC-glucan, a sample volume of 50 μL was collected from the lower chamber and replaced with an equal volume of culture medium. The fluorescence intensity was subsequently evaluated using a fluorescence microplate reader (PerkinElmer, Waltham, Massachusetts, USA).

Immunoprecipitation

Dissolve the cells in immunoprecipitation buffer (Beyotime) and combine an equal amount of 500 μg of protein with antibodies targeting DDIT4 (10638-1-AP, Proteintech) and NDUFS3 (15066-1-AP). The specific antibodies from Proteintech were gently

incubated with rotation at 4°C overnight. Subsequently, add 40 μL of Protein A&G agarose beads (Beyotime) and continue incubation for an additional 2 hours. The agarose beads were precipitated by instantaneous centrifugation at 4°C, followed by three washes with lysis buffer. The complex was eluted from the beads by heating the sample in loading buffer containing SDS, after which it was utilized for Western blotting.

Evaluation of Mitochondrial Complex 1 Activity

The activity of mitochondrial respiratory chain complex I was assessed in accordance with the kit manual (BC0515, Solarbio). Following the manufacturer's guidelines, each group of treated cells underwent ultrasonic treatment to facilitate mitochondrial isolation. Subsequently, the isolated mitochondria were mixed with the appropriate substrate solution, and the activity of complex I was quantified using spectrophotometry.

Cytoplasmic DNA enrichment

To detect mitochondrial DNA in the cytoplasm, brain microvascular endothelial cells were seeded in 6-well plates at a density of 10^6 cells per well. The cells were incubated on ice with 1% NP-40 for 15 minutes. Following incubation, the supernatant was collected and centrifuged at 16,000 g for 15 minutes at 4°C. The resulting supernatant was transferred to a new 1.5 mL EP tube, and DNA extraction was performed using the TIANamp genomic DNA kit. The level of mitochondrial DNA was quantified by qPCR; specifically, the ratio of mitochondrial DNA to nuclear DNA was determined through

real-time fluorescence quantitative PCR. The primer sequences utilized are presented in Table S3.

Mitochondrial isolation

Mitochondria were isolated using the Mitochondrial/Cytoplasmic Isolation Kit (ab65320). In brief, 5 x 10^6 primary brain microvascular endothelial cells were digested from a 10 cm tissue culture dish utilizing 1X PBS-EDTA and centrifuged at 600 rcf for 5 minutes at 4 °C. The cells were then suspended in 500 μL of the provided cytoplasmic extraction buffer mixture (without DTT or protease inhibitors) and incubated on ice for 10 minutes. A tightly fitted glass homogenizer was employed to homogenize the cell suspension on ice by performing approximately 100 strokes to lyse the cells, followed by differential centrifugation. The resulting homogenate was subjected to centrifugation at 700 rcf for 10 minutes at 4 °C to eliminate unlysed cells and nuclei. The supernatant was carefully collected and transferred into a new EP tube. Subsequently, it was centrifuged at 10,000 rcf for an additional 30 minutes at 4 °C. The supernatant was then collected into a fresh tube, where it was supplemented with a final concentration of 4X sample buffer and 5 mM DTT. Following these preparations, proteomics analysis was conducted.

Immunoprecipitation and Tandem Mass Spectrometry Analysis

Proteins from BMECs were harvested for immunoprecipitation using the anti-DDIT4

antibodies and subjected to sodium dodecyl sulfate-polyacrylamide gel electrophoresis. The gel was stained with Coomassie blue. The lysates were digested with trypsin and subjected to liquid chromatography-tandem mass spectrometry (LC-MS/MS) analysis. LC-MS/MS analysis was performed using an Orbitrap Fusion mass spectrometer (Thermo Fisher Scientific) coupled with an EASY-nLC 1000 UPLC system (Thermo Fisher Scientific), following the manufacturer's instructions.

Transcriptome sequencing

The transcriptome sequencing experiments of microvascular tissues were completed by Wuhan GeneRead Biotechnology Co., Ltd. The starting RNA for library construction is total RNA, with a total amount >=800 ng. The library construction kit used in library construction isVAHTS Universal V10 RNA-seq Library Prep Kit. After the library passes quality control, different libraries are pooled according to effective concentration and target data volume requirements for Illumina NovaSeq X Plus sequencing, and produce 150bp paired-end reads. The basic principle of sequencing is sequencing by synthesis (Sequencing by Synthesis). The image data obtained by the high-throughput sequencer is converted into sequence data (reads) by CASAVA base recognition, and the file is in fastq format, which mainly contains the sequence information of the sequenced fragments and their corresponding sequencing quality information. Download the reference genome and gene model annotation file directly from the genome website. Use HISAT2 (v2.2.1) to build an index of the reference genome, and

use HISAT2 (v2.2.1) to align paired-end clean reads with the reference genome, with parameters "–phred33 --no-mixed --no-discordant". featureCounts (v2.0.1) is used to calculate the number of reads mapped to each gene. Then calculate each gene's FPKM according to the gene's length, and calculate the number of reads mapped to that gene. For those with biological replicates, DESeq2 software (1.16.1) was used to perform differential expression analysis between two comparison groups. GO enrichment analysis of differentially expressed genes was performed using clusterProfiler (4.2.0) R software, and GO terms with corrected P values less than 0.05 were considered as significantly enriched GO terms for differentially expressed genes. ClusterProfiler R software was used to analyze the statistical enrichment of differentially expressed genes in KEGG pathways.

Figure S1

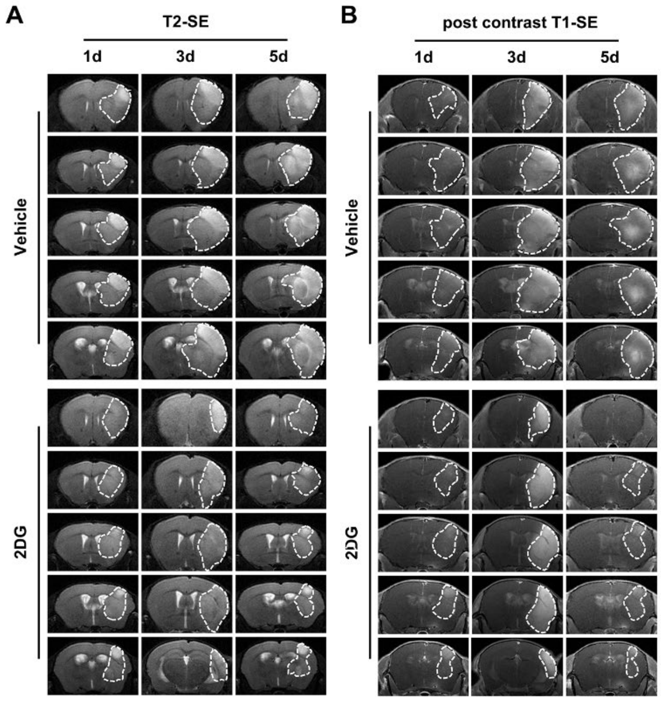

Fig S1| Multiple adjacent coronal sections of T1-weighted MRI following contrast agent administration and T2-weighted MRI A. Multiple adjacent coronal sections of T2-weighted MRI from vehicle group and 2DG treated group. **B.** Multiple adjacent coronal sections of T1-weighted MRI following contrast agent administration from vehicle group and 2DG treated group.

Figure S2

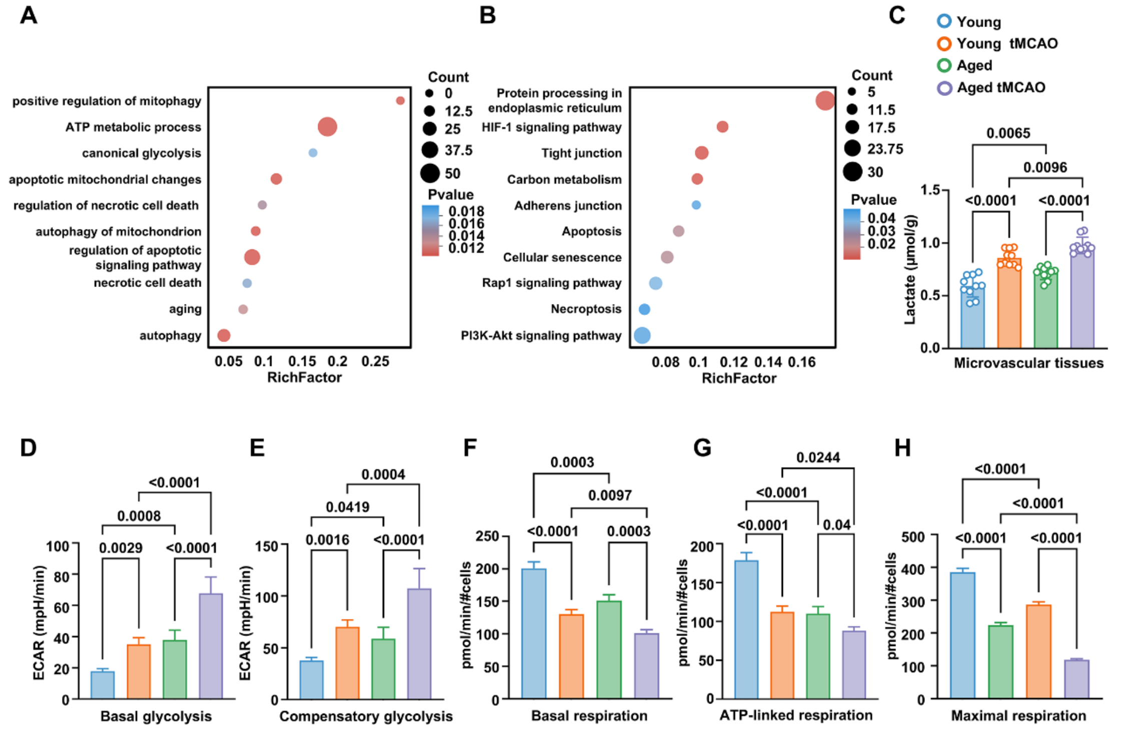

Fig S2| Single-cell sequencing reveals a stroke-induced expansion of a hyper-glycolytic endothelial subpopulation characterized by profound mitochondrial dysfunction and concomitant activation of necroptosis A, Enrichment analysis of GO pathways for differentially expressed genes in cluster 2 endothelial cells, both prior to and following ischemia-reperfusion. **B,** Enrichment analysis of KEGG pathways for differentially expressed genes in cluster 2 endothelial cells, both prior to and following ischemia-reperfusion. **C,** Intracellular lactate levels of microvascular tissues isolated from 8-week-old and 20-month-old mice after tMCAO (n = 10 mice per group). **D-E,** The ECAR of BMECs isolated from aged and young mice treated with or without OGDR (n = 5 mice per group). **F-H,** The ECAR of BMECs isolated from aged and young mice treated with or without OGDR (n = 5 mice per group). Data are mean ± SEM.

Figure S3

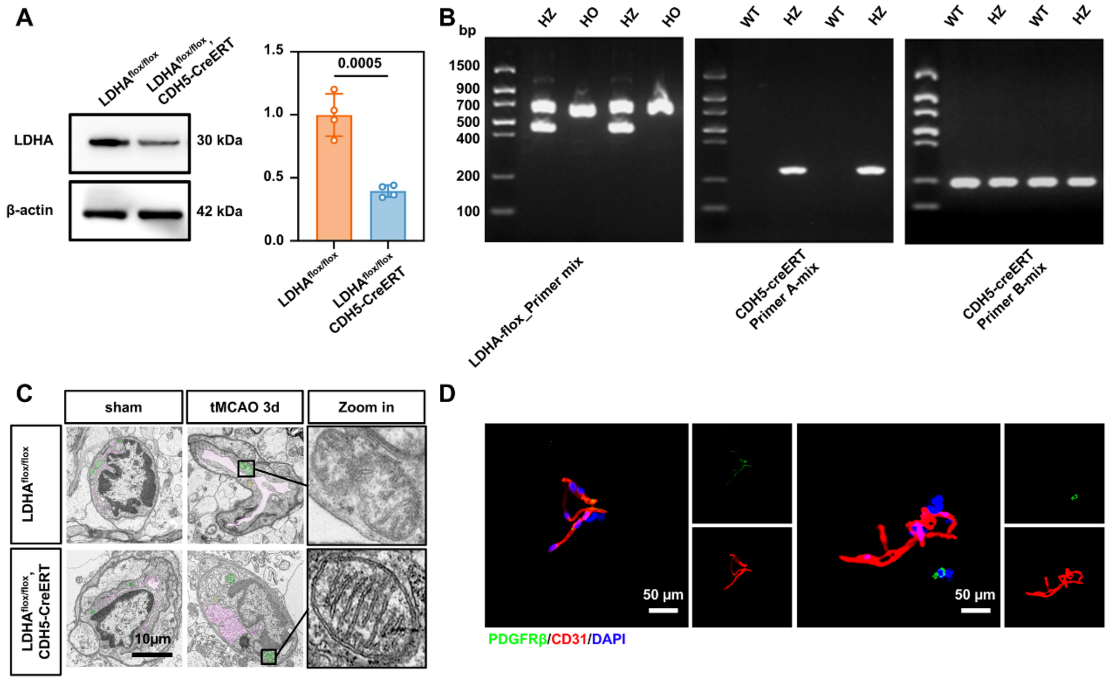

Fig S3| Conditional knockout of LDHA from BMECs alleviates BBB injury and EC death A, Verification of conditionally knockout of LDHA expression in LDHA$^{flox/flox}$; CDH5-CreERT mice (n = 3 per group). **B,** Genotyping of LDHA$^{flox/flox}$ mice and CDH5-CreERT mice. **C,** Electron microscopy of endothelial cells at the blood-brain barrier demonstrated that following LDHA knockout, the structural alterations in the mitochondria of endothelial cells were mitigated. **D,** The purity of the resulting microvessels was consistently verified by immunofluorescence staining, showing strong positivity for endothelial markers (CD31) and negativity for the pericyte marker PDGFRβ. Data are mean ± SEM.

Figure S4

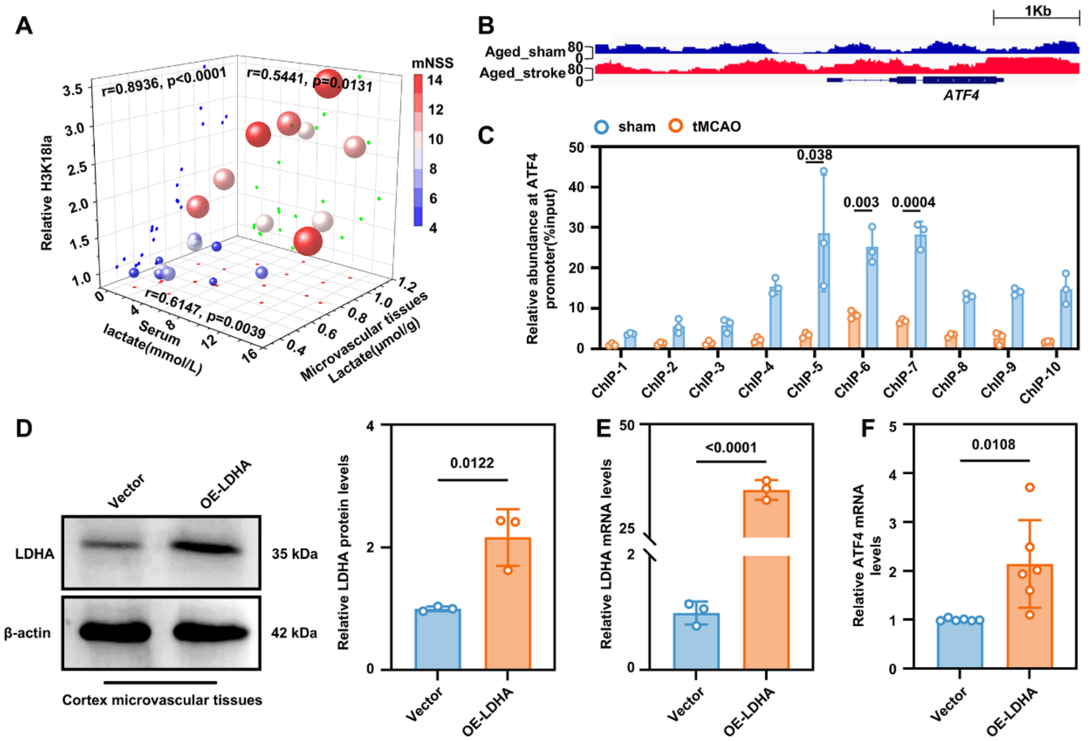

Fig S4| ATF4 is transcriptionally regulated by H3K18la A, Correlation analysis of serum lactate levels with H3K18la and lactate levels in microvascular tissues. **B,** Genome browser tracks of CUT&Tag signal at the representative target gene loci. **C,** qChIP analysis of the indicated promoters was performed using antibodies against H3K18la in microvascular tissues from 20-month-old sham and tMCAO mice (n = 3 mice per group). **D-E,** Western blotting and quantitative PCR (qPCR) were employed to assess the overexpression of LDHA mediated by transfection plasmids in primary brain microvascular endothelial cells (n = 3 per group). **F,** qPCR assays monitoring expression of the ATF4 in LDHA overexpression endothelial cell (n = 6 per group). Data are mean ± SEM.

Figure S5

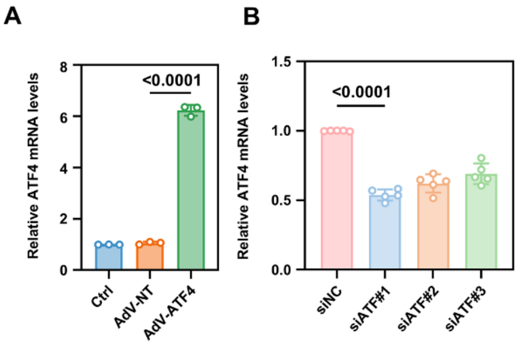

Fig S5| Verification of the regulation of ATF4 in primary BMECs *in vitro* A, Real-time fluorescence quantitative analysis indicated that the overexpression of ATF4 in primary BMECs was successfully achieved (n = 3 per group). **B,** Real-time fluorescence quantitative analysis demonstrated the successful knockdown of ATF4 in primary BMECs (n = 5 per group). Data are mean ± SEM.

Figure S6

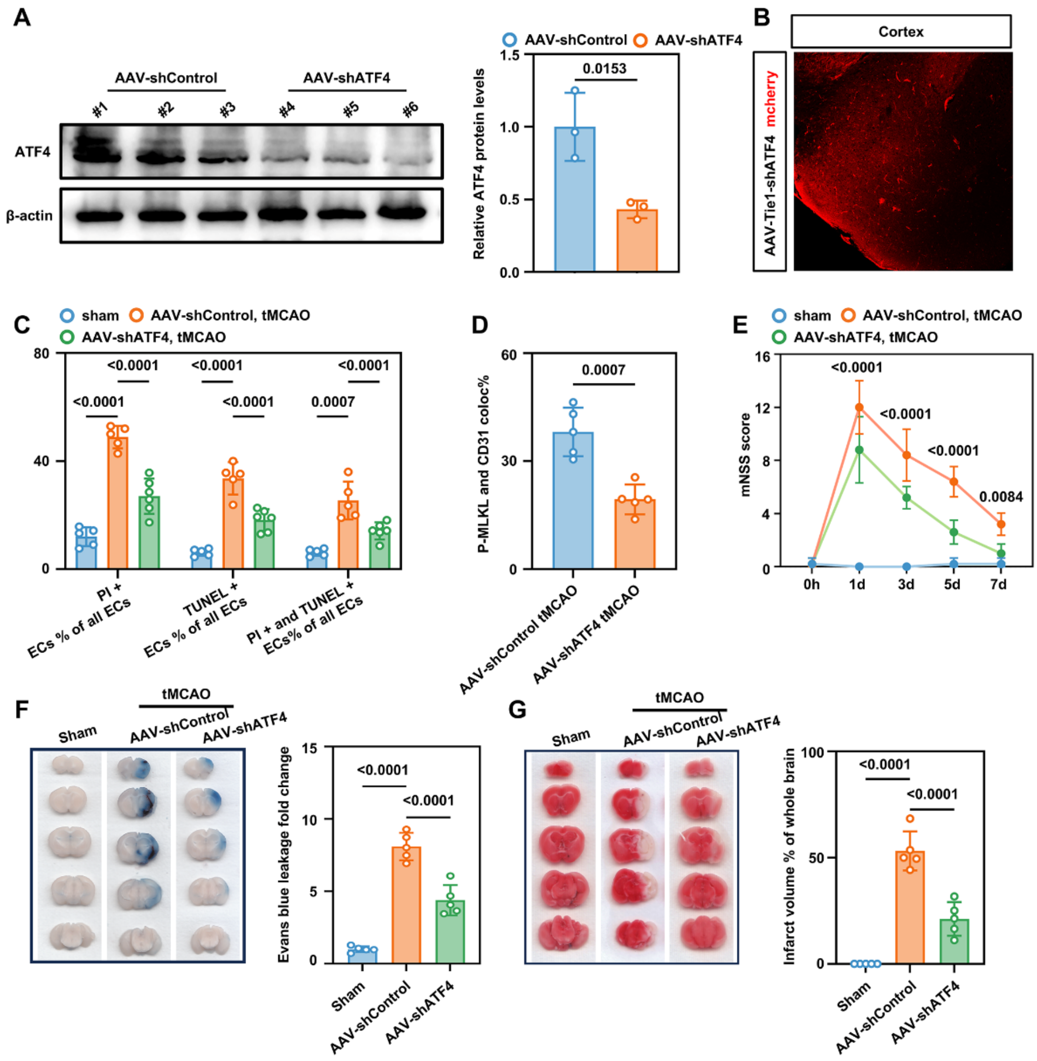

Fig S6| ATF4 knockdown reduced necroptosis in BMECs and alleviated edema and brain injury A, Western blot analysis demonstrated a successful knockdown of ATF4 in the microvascular tissues of aged mice, graphs are representative of three independent experiments. **B,** Fluorescence imaging demonstrates the successful transfection of AAV virus into microvascular endothelial cells. **C,** Statistical analysis results of immunofluorescence staining for TUNEL (green, indicating apoptosis) and propidium iodide (PI) (red, indicating necrosis) in endothelial cells within the ischemia-reperfusion cortical region following ATF4 knockdown (n = 3 per group). **D,**

Quantitative analysis of the co-localization of p-MLKL and endothelial cells (n = 5 per group). **E,** Modified neurological severity score from day 0 to day 7 after tMCAO in different groups (n = 5 per group). **F,** Evans blue leakage of aged mice brains in coronal sections and extravasation (fold change relative to sham) from Sham, AAV-shControl tMCAO, and AAV-shATF4 tMCAO groups (n = 5 per group). **G,** Representative images and statistical results of TTC (n = 5 per group). Data are mean ± SEM.

Figure S7

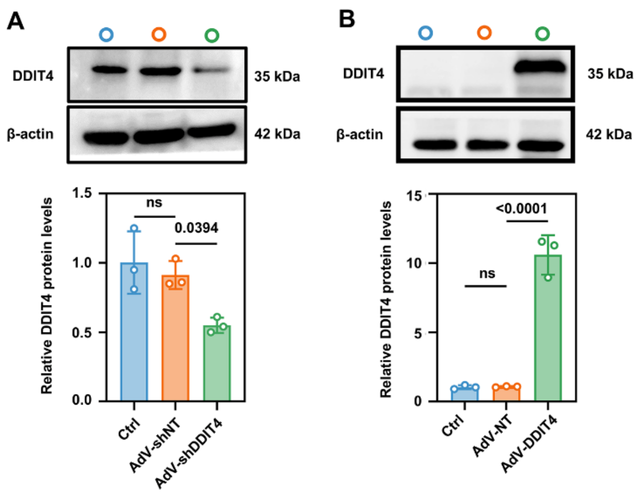

Fig S7| Verification of the regulation of DDIT4 in primary BMECs *in vitro* A, Western blotting and quantitative analysis showed successful endogenous knockdown of DDIT4 in primary BMECs (n = 3 per group). **B,** Western blot analysis demonstrated a successful overexpression of DDIT4 in primary endothelial cells (n = 3 per group). Data are mean ± SEM.

Figure S8

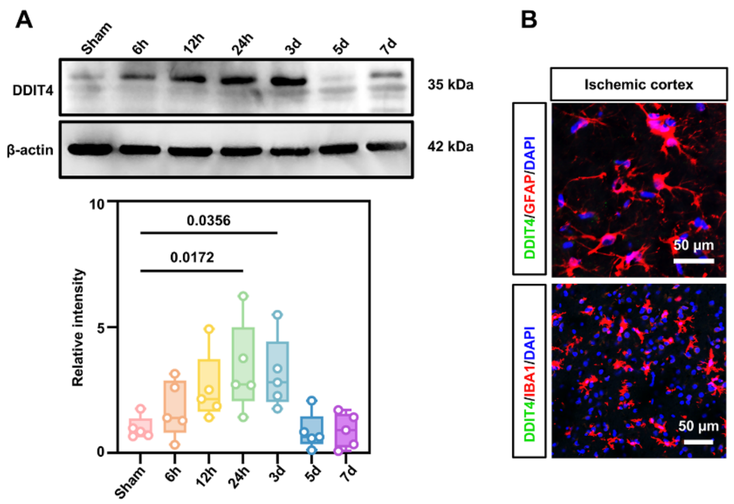

Fig S8| DDIT4 is upregulated in BMECs of tMCAO mice A, Western blot analysis demonstrated the time course of DDIT4 expression after ischemia-reperfusion (n = 5 per group). **B,** Representative images illustrating the co-staining of DDIT4 with astrocytes (anti-GFAP) and microglia (anti-IBA1) in the ischemia-reperfusion region. Data are mean ± SEM.

Figure S9

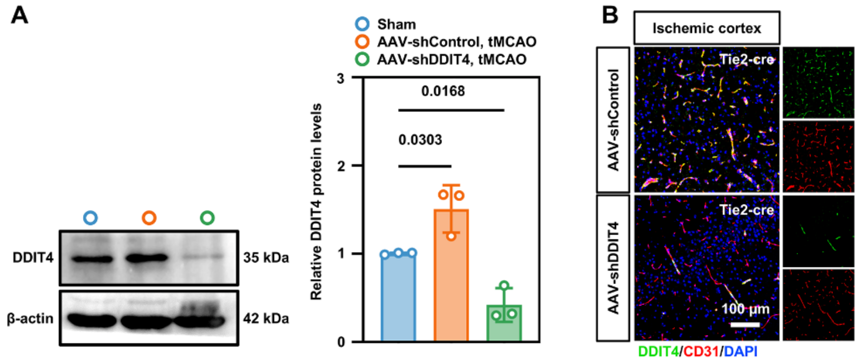

Fig S9| The verification of DDIT4 knockdown *in vivo* A, Western blot analysis demonstrated a successful knockdown of DDIT4 in the cerebral microvascular tissues of tMCAO mice (n = 3 per group). **B,** Fluorescence imaging demonstrated DDIT4 was knocked down in BMECs. Data are mean ± SEM.

Figure S10

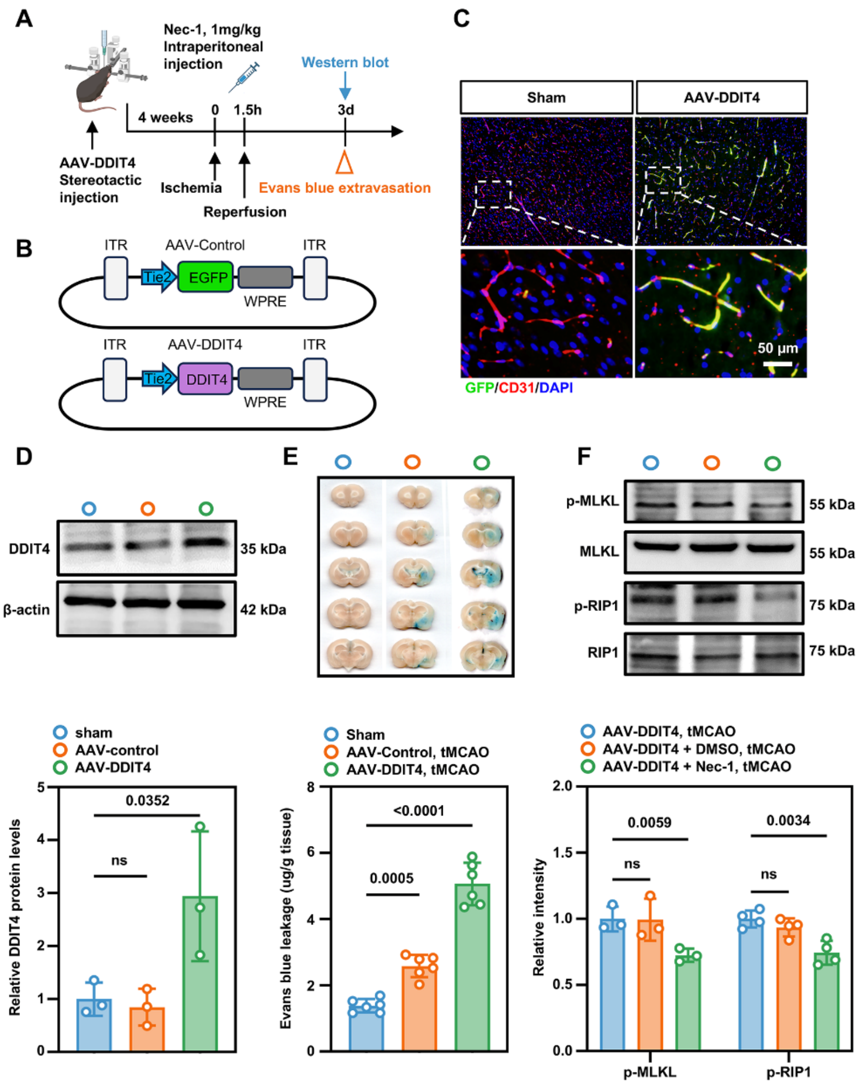

Fig S10| Specific overexpression of DDIT4 promotes EC necroptosis and BBB injury A, Experimental design and timeline of ischemic stroke, DDIT4 overexpression, and analysis in mice. **B,** Schematic diagram of the AAV used for DDIT4 overexpression *in vivo*. **C,** Fluorescence imaging demonstrates the successful transfection of AAV virus into microvascular endothelial cells. **D,** Western blot analysis demonstrated a successful overexpression of DDIT4 in cerebral microvascular tissues (n = 3 per group). **E,** Evans blue leakage of mice brains in coronal sections and extravasation (μg/g tissue) from Sham, AAV-control tMCAO, and AAV-DDIT4 tMCAO groups (n = 6 per group). **F,**

Expression levels of p-RIP1/RIP1 and p-MLKL/MLKL proteins in cerebral microvascular tissues of DDIT4 overexpression mice after tMCAO with or without Nec-1 treatment (n = 3-4 per group). Data are mean ± SEM.

Figure S11

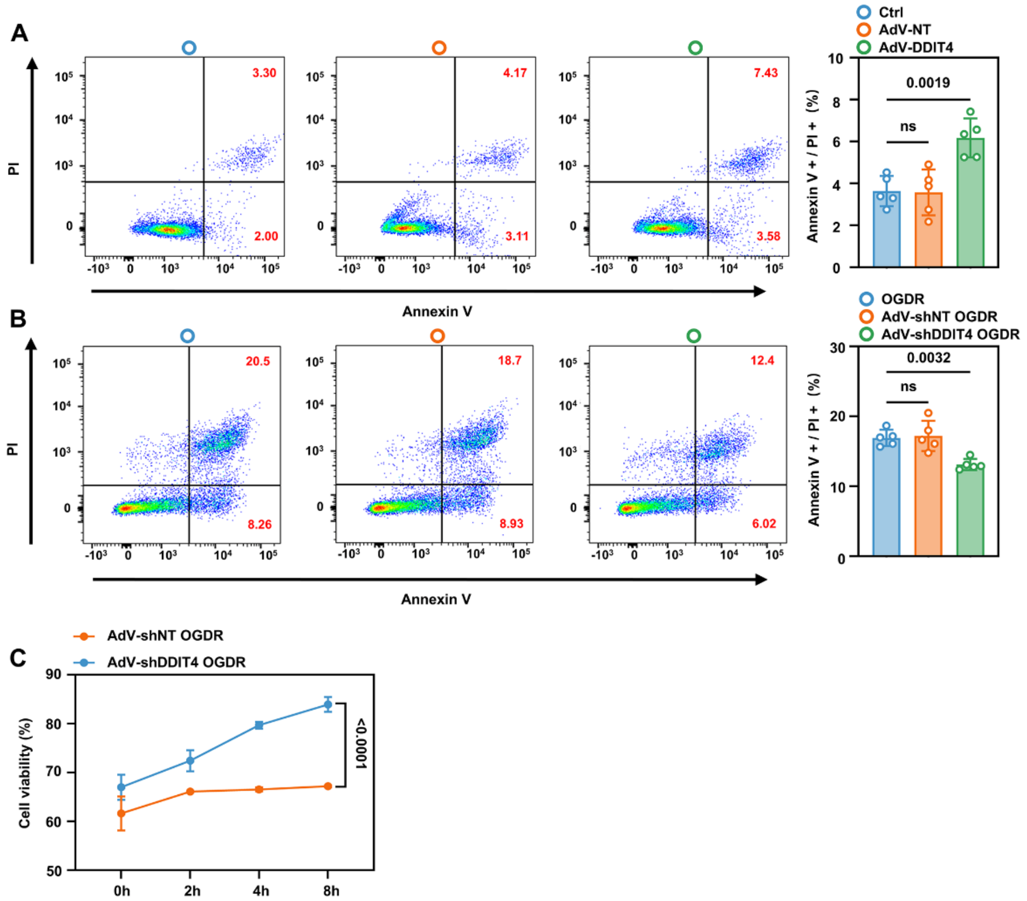

Fig S11| Knockdown of DDIT4 decreases necroptosis of primary BMECs following OGDR treatment *in vitro* A-B, Representative flow cytometry images of ECs stained with PI/annexin V. The necroptosis activation was represented by ratio of PI + /annexin V + (n = 5 per group). **C,** CCK-8 results of cell viability of primary microvascular endothelial cells (n = 3 independent experiments). Data are mean ± SEM.

Figure S12

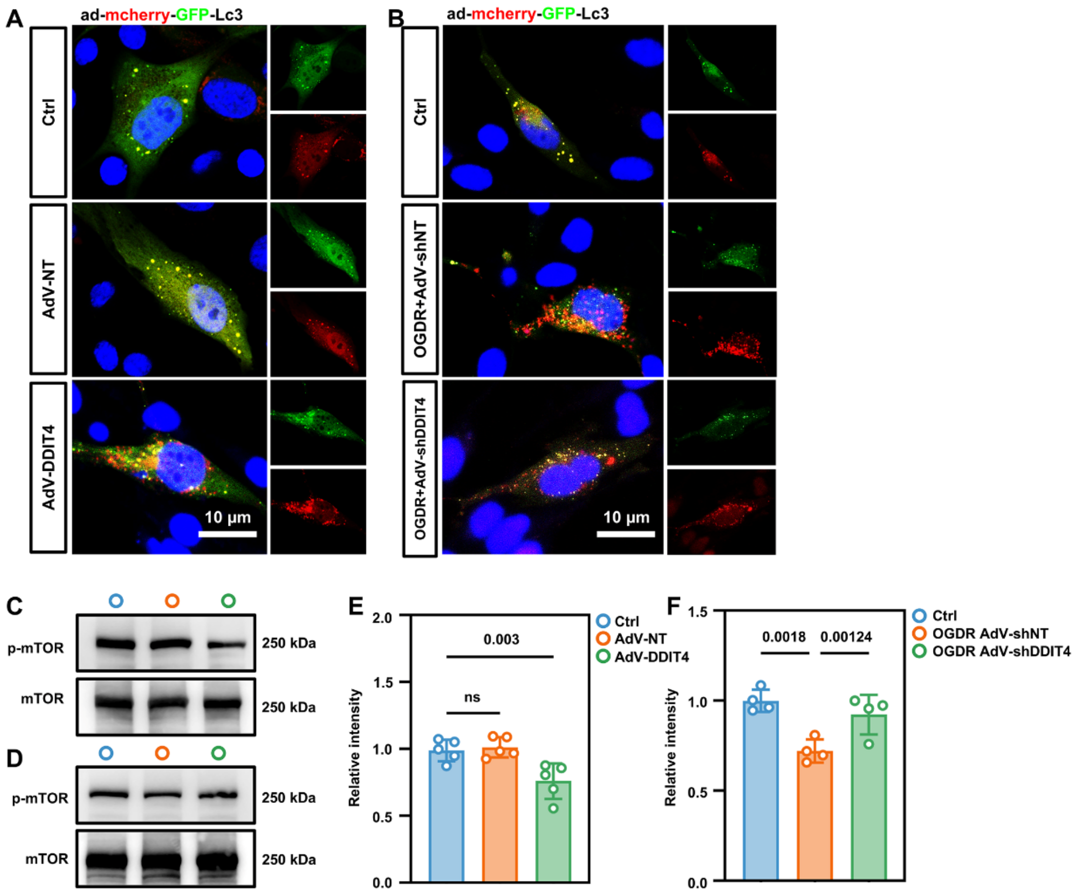

Fig S12| DDIT4 promotes autophagy in BMECs A, Immunofluorescence images for BMECs transfected with ad-mCherry-eGFP-Lc3 and then treated with AdV-NT, AdV-DDIT4. DAPI was used to visualize nuclei (blue). **B,** Immunofluorescence images for BMECs transfected with ad-mCherry-eGFP-Lc3 and then treated with AdV-shNT, AdV-shDDIT4 following OGDR. DAPI was used to visualize nuclei (blue). **C-D,** Western blotting of p-mTOR/mTOR in BMECs with indicated treatments. **E-F,** Expression levels of p-mTOR/mTOR proteins in BMECs with indicated treatments (n = 4-5 per group). Data are mean ± SEM.

Figure S13

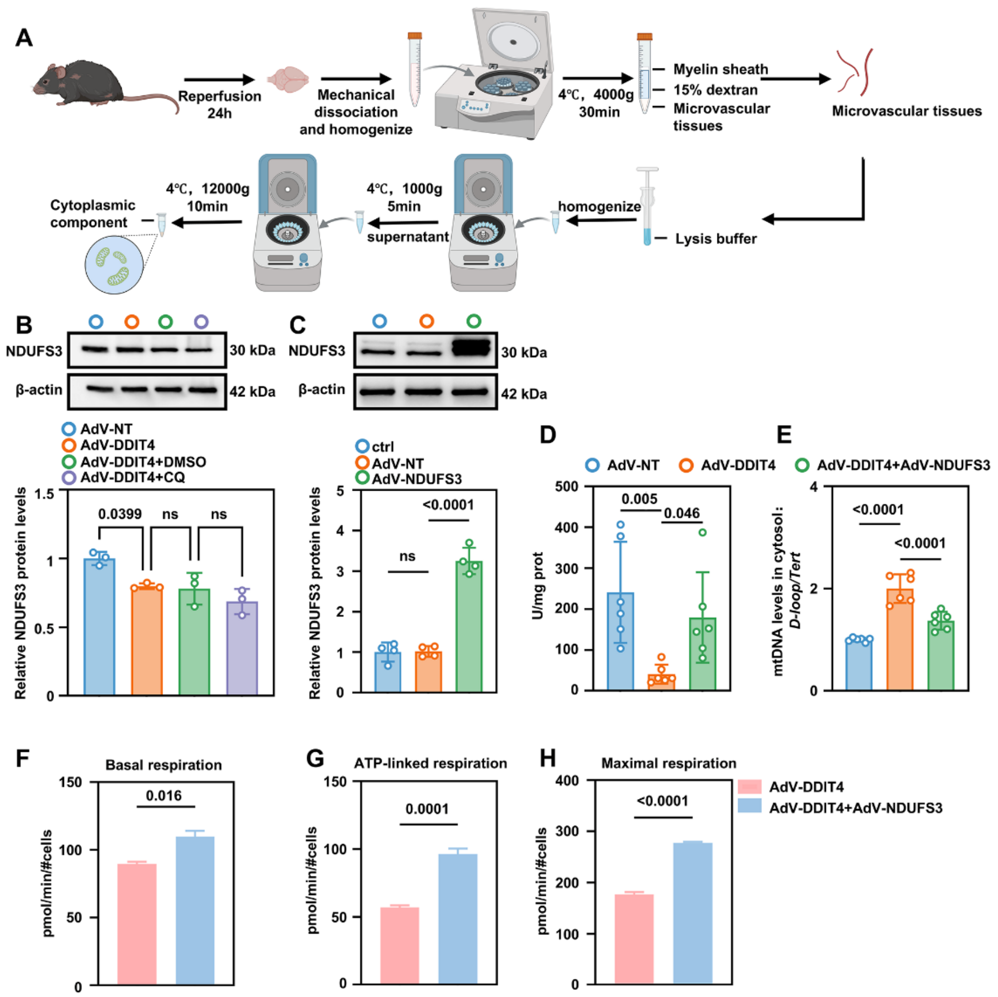

Fig S13| Mitochondrial dysfunction induced by DDIT4 can be ameliorated through the restoration of NDUFS3 expression A, Workflow diagram for mitochondrial extraction from cerebral microvascular tissues. **B,** Western blotting of NDUFS3 in indicated cells. Endothelial cell was treated with CQ 6h for harvest. And the quantification of protein was shown in below (n = 3 per group). **C,** Western blotting was employed to confirm the overexpression of NDUFS3 in primary brain microvascular endothelial cells (n = 3 per group). **D,** Determination of the activity of Mitochondrial complex I/NADH-CoQ reductase in indicated cells (n = 6 per group). **E,** qPCR of mtDNA (*D-loop*) from cytosolic fractions, quantified relative to total nuclear

DNA (*Tert*) in indicated cells (n = 6 per group). **F-H,** Basal respiration, ATP-linked respiration, and maximal respiration in primary BMECs. Data are mean ± SEM.

Figure S14

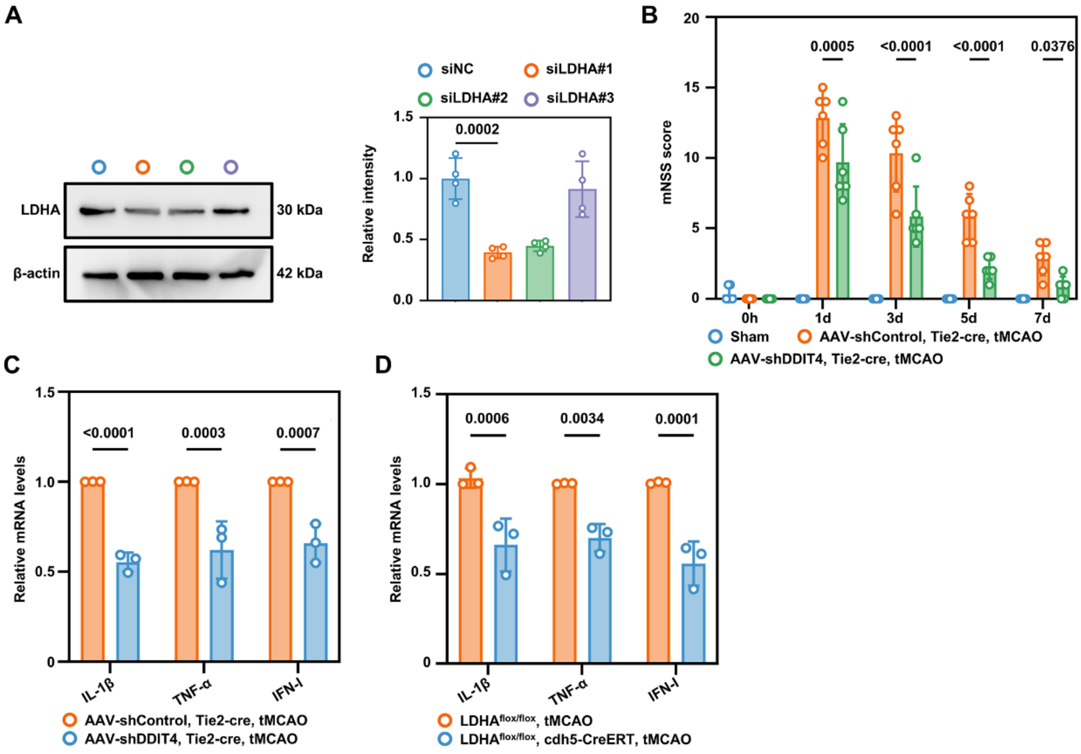

Fig S14| Interruption of glycolysis/H3K18la/ATF4-DDIT4 feedback loop of BMECs reduces brain injury and inflammatory *in vivo* A, Western blotting of LDHA in primary BMECs treated with or without siRNA. The quantification of protein was shown in the right panel (n = 4 per group). **B,** Modified neurological severity score at 0, 1, 3, 5, 7 days after tMCAO in different groups (n = 6 per group). **C,** qPCR analysis of pro-inflammation-related genes in microvascular tissues isolated from AAV-shControl; Tie2-cre; tMCAO and AAV-shControl; Tie2-cre; tMCAO mice (n = 3 mice per group). **D,** qPCR analysis of pro-inflammation-related genes in microvascular tissues isolated from LDHA$^{flox/flox}$; tMCAO and LDHA$^{flox/flox}$; CDH5-CreERT; tMCAO mice (n = 3 mice per group). Data are mean ± SEM.

Table S1. Baseline characteristics of patients

Variables	Total (n = 181)	Age ≤ 54 (n = 51)	55≤age≤74 (n = 68)	age≥75 (n = 62)
Male, n(%)	116 (64.09)	40 (78.43)	45 (66.18)	31 (50.00)
Hypertension, n(%)	92 (50.83)	25 (49.02)	28 (41.18)	39 (62.90)
Diabetes, n(%)	45 (24.86)	8 (15.68)	15 (22.06)	22 (35.48)
Coronary heart disease, n(%)	17 (9.39)	3 (5.88)	6 (8.82)	8 (12.90)
Renal dysfunction, n(%)	20 (11.05)	2 (3.92)	5 (7.35)	13 (20.97)
Admission NIHSS score, M (Q_1, Q_3)	4.00 (2.00, 7.00)	2.00 (1.00,5.00)	4.00 (2.00,6.00)	5.00 (2.00,12.00)
History of intracerebral hemorrhage, n (%)	5 (2.76)	2 (3.92)	1 (1.47)	2 (3.22)
Lactic acid, M (Q_1, Q_3)	4.63 (1.91, 9.50)	4.16 (1.92,8.65)	3.45 (1.87,7.43)	5.46 (1.99,11.53)

Table S2. Key resources table

REAGENT or RESOURCE	SOURCE	IDENTIFIER
**Antibodies**		
Rabbit monoclonal anti-MLKL（phospho S345）	Abcam	Cat# ab196436
Rabbit monoclonal anti-MLKL	Cell Signaling Technolgy	Cat# 37705
Rabbit monoclonal anti-RIP1（phospho S166）	Cell Signaling Technolgy	Cat# 65746
Rabbit polyclonal anti-RIP1	Proteintech	Cat# 17519-1-AP
Goat polyclonal anti-Iba1	Abcam	Cat# ab5076
Mouse Polyclonal anti-GFAP	Proteintech	Cat# 60190-1-AP

Goat Polyclonal anti-CD31	R&D systems	Cat# AF3628
Sheep polyclonal anti-Fibrinogen	Abcam	Cat# ab118533
Rabbit monoclonal anti-GLUT1	Abcam	Cat# ab115730
Rabbit monoclonal anti-LC3B	Abcam	Cat# ab192890
Mouse monoclonal anti-TOMM20	Abcam	Cat# ab56783
Goat Anti-Type IV Collagen	SouthernBiotech	Cat# 1340-01
Lycopersicon Esculentum (Tomato) Lectin (LEL, TL), DyLight® 594	VectorLaboratories	Cat# DL-1177-1
Rabbit monoclonal anti-DDIT4	Abcam	Cat# ab191871
Rabbit polyclonal anti-DDIT4	Proteintech	Cat# 10638-1-AP
Rabbit monoclonal anti-mTOR（phospho S2448）	Cell Signaling Technolgy	Cat# 5536
Mouse monoclonal anti-mTOR	Proteintech	Cat# 66888-1-Ig
Rabbit polyclonal anti-NDUFS3	Proteintech	Cat# 15066-1-AP
Rabbit monoclonal anti-Ubiquitin	Cell Signaling Technolgy	Cat# 43124
Rabbit monoclonal anti-ATF4	Cell Signaling Technolgy	Cat# 11815
Mouse monoclonal anti-ATF4	Proteintech	Cat# 60035-1-Ig
Rabbit polyclonal anti-LDHA	Proteintech	Cat# 19987-1-AP
Rabbit polyclonal anti-Pan Kla	PTM BIO	Cat# PTM-1401
Rabbit monoclonal anti-H3K9la	PTM BIO	Cat# PTM-1419
Rabbit monoclonal anti-H3K14la	PTM BIO	Cat# PTM-1414
Rabbit monoclonal anti-H3K18la	PTM BIO	Cat# PTM-1416
Rabbit monoclonal anti-H3K18la	PTM BIO	Cat# PTM-1427
Rabbit monoclonal anti-H4K5la	PTM BIO	Cat# PTM-1407
Rabbit monoclonal anti-H4K8la	PTM BIO	Cat# PTM-1415
Rabbit monoclonal anti-H4K12la	PTM BIO	Cat# PTM-1411
Rabbit monoclonal anti-H4K16la	PTM BIO	Cat# PTM-1417

Rabbit monoclonal anti-H3	PTM BIO	Cat# PTM-1001
Rabbit monoclonal anti-H4	PTM BIO	Cat# PTM-1015
Rabbit monoclonal anti-β-actin	Abclonal	Cat# AC026
HRP-conjugated Goat anti-Rabbit IgG	Abclonal	Cat# AS014
HRP-conjugated Goat anti-Mouse IgG	Abclonal	Cat# AS003
Alexa Fluor 594 AffiniPure donkey anti-goat IgG	ThermoFisher	Cat# A11058
Alexa Fluor 647 AffiniPure donkey anti-goat IgG	ThermoFisher	Cat# A21447
Alexa Fluor 488 AffiniPure donkey anti-rabbit IgG	ThermoFisher	Cat# A21206
Alexa Fluor 488 AffiniPure donkey anti-goat IgG	ThermoFisher	Cat# A11055
Alexa Fluor 594 AffiniPure donkey anti-mouse IgG	ThermoFisher	Cat# A21203
Alexa Fluor 647 AffiniPure donkey anti-sheep IgG	ThermoFisher	Cat# A21448
Alexa Fluor 488 AffiniPure donkey anti-sheep IgG	ThermoFisher	Cat# A11015
**Chemicals, peptides, and recombinant proteins**		
FITC-Dextran	Sigma	Cat# FD40S
Propidium Iodide	MCE	Cat# 25535-16-4
2-Deoxy-D-glucose	MCE	Cat# 154-17-6
Dextran T70	Shanghai yuanye Bio-Technology Co., Ltd	Cat# S14112
Evans Blue	Sigma	Cat# E2129
MG132	Selleck	Cat# S2619

Z-DEVD-FMK	Selleck	Cat# S7312
Chloroquine	Sigma	Cat# C6628
Tamoxifen	Beyotime	Cat# Y258299
**Critical commercial assays**		
One Step TUNEL Apoptosis Assay Kit	Beyotime	Cat# C1088
Seahorse XF Cell Mito Stress Test Kit	Agilent Technologies	Cat# 103015-100
Seahorse XF Glycolytic Rate Assay Kit	Agilent Technologies	Cat# 103344-100
Adult Brain Dissociation Kit	Miltenyi Biotec	Cat# 130-107-677
anti-CD31-coated MicroBeads	Miltenyi Biotec	Cat# 130-097-418
Lactic Acid(LA) Content Assay Kit	Solarbio	Cat# BC2235
Pyruvate(PA) Content Assay Kit	Solarbio	Cat# BC2205
Mitochondrial complex I/NADH-CoQ reductase Activity Assay Kit	Solarbio	Cat# BC0515
SimpleChIP® Enzymatic Chromatin IP Kit (Magnetic Beads)	Cell Signaling Technolgy	Cat# 9003S
TTC	Servicebio	Cat# G1017
MitoTracker™ Red CMXRos	ThermoFisher	Cat# M7512
MitoSox Red	ThermoFisher	Cat# M36007
Enhanced mitochondrial membrane potential assay kit with JC-1	Beyotime	Cat# C2003S
Annexin V-FITC Apoptosis Detection Kit	Beyotime	Cat# C1062L
CCK-8 Cell Counting Kit	Vazyme	Cat# A311
mCherry-eGFP-LC3	HANBIO	Cat# HB-AP210
Immunoprecipitation Kit with Protein A+G Agarose Gel	Beyotime	Cat# P2197M
Cell Mitochondria Isolation Kit	Beyotime	Cat# C3601

Table S3. Primers Used for qRT-PCR Analysis

Gene	Forward	Reverse
Mouse ATF4	GCCTGACTCTGCTGCTTATATTACTC	CAGGTAGGACTCAGGGCTCATAC
Mouse DDIT4	CCGAGCTTGTTCGTCGTTTG	AACACCCACCCCCTTCCTAT
Mouse SESN2	ACACCCCGACTACCTTAGCA	AACTCGGTCATGTGGGAACC
Mouse NOXA	GTGGAGTGCACCGGACATAA	GGGCTTCTTCTCATCGTGCT
Mouse BCL2	GAACTGGGGGAGGATTGTGG	GGGGTGACATCTCCCTGTTG
Mouse CHOP	AAGGAGAAGGAGCAGGAGAATGAG	GAGCCCGCCGTGTGGTC
Mouse LC3B	AGCCTTCTTCCTCCTGGTGAATG	CTCTCTCGCTCTCGTACACTTCAG
Mouse Atg3	ATGAGCAACGGCAGCCTTTAAC	TAGGAGGTGGTGGGAGGTGAG
Mouse Atg7	AGCAGTGATGACCGCATGAATG	CAGGCTGACAGGAAGGACATTATC
Mouse Atg12	CCTGCTGAAGGCTGTAGGAGAC	CTGATGAAGTCGATGAGTGCTTGG
Mouse NDUFS3	TGGCAGCACGTAAGAAGGG	CTTGGGTAAGATTTCAGCCACAT
Mouse Nuclear Tert	CTAGCTCATGTGTCAAGACCCTCTT	GCCAGCACGTTTCTCTCGTT
Mouse mtDloop	CCCTTCTTTATTTGGTCT	TGGTTTCACGGAGGATGG

Mouse TNFα	TAGCCCACGTCGTAGCAAAC	TGTCTTTGAGATCCATGCCGT
Mouse IL1β	TGCCACCTTTTGACAGTGATG	TGATGTGCTGCTGCGAGATT

Table S4. Primers Used for qChIP

Gene	Forward	Reverse
DDIT4 promoter-1	CCTAGTTGGAGGAATGGGTGC	TTCCGTAAGAGCAGGTGACA
DDIT4 promoter-2	GTTCCAGAAGCAAACACGGC	GTGGGAGACCCGCTCATTTAT
DDIT4 promoter-3	TGTGGGAGACCTCGGATTCA	GCAACATAGCTCCTACCACTGAT
DDIT4 promoter-4	GCCTGACTTCCCTGACAAGTG	CAAACCAGATCAGACAGGCAAG
DDIT4 promoter-5	ACAATCGCTATCAGCCCTCG	GTGCAATAGCCATCAGGGGT
DDIT4 promoter-6	CATTGAATCCTCCCCCGACC	TCAAACCGACACACTGACCC
DDIT4 promoter-7	ATGGACCAGGCGCTATGAAT	ACATACATCACTGCTCGCCT
DDIT4 promoter-8	GGGACAACGGAGTAACGTCT	CCAAGAGCCAAGGACACTACC
DDIT4 promoter-9	GTGTAGTCGTAGGCTGTCCC	TTTCTGGAAGGGATCCGAGG
DDIT4 promoter-10	CCGGTGCCTATTGGATGAAGT	GCAGCCTATAAGGGCTCTCC
ATF4 promoter-1	TGGTGTTGGAGGTCCCTTTTAT	GTCTTGAGTCCCCCACTCTTC
ATF4 promoter-2	GCCATGTGGAAGGTTTCTGG	ATGCTGAATTTGTAACCCCGTG
ATF4 promoter-3	ACTGTGGCTCCTGTTTCTGTTT	AATAACGCACAAGGCCTACATA
ATF4 promoter-4	TCATGGAGTTAGGGAGAGAGGG	GCCAAGTACTCCAGTACACACA
ATF4 promoter-5	CACTGTGCCAATTGAGTGGT	GAAGCCCCAAGTACTGGTCAA
ATF4 promoter-6	CAGGATGACTTCTTGGTAAGGCT	GCCCATCACTGCTGGTTTGT
ATF4 promoter-7	GTCTCAAAGTGCGGTCCCTG	CAAATGGGCAGAGCTTTAGGC
ATF4 promoter-8	GCCGGAGAGAGCAACTACAT	TATCGGGAAGAGCCACCAGA
ATF4 promoter-9	GGTTAGGTGTCTCTCCACGC	GGTTTGCGACCAACTGAAGTC
ATF4 promoter-10	GTAACGCCCCAGTTACTCCC	GACGCAGTACACCCCGTATC

Table S5. Primers used for genotyping of murine strains

Primers	Sequence	Product size
Tek-Cre	AAAAATCAGCATTTTCAACAAA	Mutant: 179bp
	GTTTATTTACCGCCGTGTGTG	
CDH5-CreERT	CACTGGGTCCTGATGGTGCCTATC	Mutant: 248bp
	TCCTGTTGTTCAGCTTGCACCAG	
	CACTGGGTCCTGATGGTGCCTATC	WT: 199bp
	AATCCAGTCTCTCTTTTGGCGATG	
LDHA-flox	CTGAGCACACCCATGTGAGA	WT: 458bp
	AGCAACACTCCAAGTCAGGA	Mutant: 700bp

Table S6. Sequence of siRNAs

Gene	Sequence
ATF4 siRNA-1	CUAGGUCUCUUAGAUGACUAUTT
	AUAGUCAUCUAAGAGACCUAGTT
ATF4 siRNA-2	CCUCUAGUCCAAGAGACUAAUTT
	AUUAGUCUCUUGGACUAGAGGTT
ATF4 siRNA-3	CCAGAGCAUUCCUUUAGUUUATT
	UAAACUAAAGGAAUGCUCUGGTT
LDHA siRNA-1	AAGAUAAGCUAAAGGGAGATT
	UCUCCCUUUAGCUUAUCUUTT
LDHA siRNA-2	UCAAUUUGGUCCAGCGAAATT
	UUUCGCUGGACCAAAUUGATT
LDHA siRNA-3	UGAUCAAGCUGAAAGGUUATT
	UAACCUUUCAGCUUGAUCATT
